# Effect of regional crosstalk between sympathetic nerves and sensory nerves on temporomandibular joint osteoarthritic pain

**DOI:** 10.1038/s41368-024-00336-6

**Published:** 2025-01-07

**Authors:** Zhangyu Ma, Qianqian Wan, Wenpin Qin, Wen Qin, Janfei Yan, Yina Zhu, Yuzhu Wang, Yuxuan Ma, Meichen Wan, Xiaoxiao Han, Haoyan Zhao, Yuxuan Hou, Franklin R. Tay, Lina Niu, Kai Jiao

**Affiliations:** 1 Department of Stomatology, Tangdu Hospital & State Key Laboratory of Oral & Maxillofacial Reconstruction and Regeneration, School of Stomatology, The Fourth Military Medical University, Xi’an, China; 2 State Key Laboratory of Oral & Maxillofacial Reconstruction and Regeneration & National Clinical Research Center for Oral Diseases & Shaanxi Key Laboratory of Stomatology, School of Stomatology, The Fourth Military Medical University, Xi’an, China; 3 The Graduate School, Augusta University, Augusta, GA USA

**Keywords:** Oral diseases, Pathogenesis

## Abstract

Temporomandibular joint osteoarthritis (TMJ-OA) is a common disease often accompanied by pain, seriously affecting physical and mental health of patients. Abnormal innervation at the osteochondral junction has been considered as a predominant origin of arthralgia, while the specific mechanism mediating pain remains unclear. To investigate the underlying mechanism of TMJ-OA pain, an abnormal joint loading model was used to induce TMJ-OA pain. We found that during the development of TMJ-OA, the increased innervation of sympathetic nerve of subchondral bone precedes that of sensory nerves. Furthermore, these two types of nerves are spatially closely associated. Additionally, it was discovered that activation of sympathetic neural signals promotes osteoarthritic pain in mice, whereas blocking these signals effectively alleviates pain. In vitro experiments also confirmed that norepinephrine released by sympathetic neurons promotes the activation and axonal growth of sensory neurons. Moreover, we also discovered that through releasing norepinephrine, regional sympathetic nerves of subchondral bone were found to regulate growth and activation of local sensory nerves synergistically with other pain regulators. This study identified the role of regional sympathetic nerves in mediating pain in TMJ-OA. It sheds light on a new mechanism of abnormal innervation at the osteochondral junction and the regional crosstalk between peripheral nerves, providing a potential target for treating TMJ-OA pain.

## Introduction

Temporomandibular joint disorder (TMD) is one of the most common disorders in the orofacial region, with ~33% of the population exhibiting at least one symptom of TMD.^1,2^ Among its manifestations, temporomandibular joint osteoarthritis (TMJ-OA), a late-stage progressive form of TMD, often accompanies chronic pain. The debilitating pain associated with TMJ-OA severely affects patients’ physical and mental well-being, and constitutes a primary medical burden within the chronic disease arena.^3^ This pain also limits functional recovery and increases the risk of disability among TMJ-OA patients. However, due to the unclear mechanisms underlying TMJ-OA related pain, there is a lack of effective therapies. Elucidating the pathological mechanisms of TMJ-OA pain and developing effective treatments are crucial to alleviating patient suffering.

The pain associated with osteoarthritis is modulated by both the central and peripheral nervous systems.^4,5^ Abnormal distribution of nerve endings and increased sensitivity of regional sensory nerves are the main sources of osteoarthritic pain.^6,7^ Osteoarthritic changes involve the joint capsule, synovium, subchondral bone, fat pad, ligaments, and other tissues.^6,8–10^ The relationship between inflammation of the synovium and osteoarthritis-related pain has been extensively investigated.^11–13^ However, it often occurs that pain precedes the development of synovitis in the early stages of osteoarthritis, or even appears in the absence of synovitis.^6^ Osteoarthritis patients experience rapid and significant pain relief after knee arthroplasty. This procedure involves the removal of subchondral bone and the joint cartilage covering the subchondral bone surface.^14,15^ Because articular cartilage typically lacks innervation and cannot generate pain,^16^ this evidence suggests that the subchondral bone is the primary source of osteoarthritis-related pain. In osteoarthritis, abnormal remodeling of subchondral bone and formation of osteophytes are often accompanied by neurovascularization.^17,18^ Previous studies have suggested that netrin-1, secreted by osteoclasts during abnormal subchondral bone remodeling, may induce sensory nerve innervation and osteoarthritis-related pain through its DCC (deleted in colorectal cancer) receptor.^9^ During bone remodeling, the level of prostaglandin E2 (PGE2) also increases in the subchondral bone. This can activate various pain-related ion channels through the receptor EP4 (prostaglandin E2 receptor subtype 4) expressed on sensory nerves, enhancing the excitability of sensory neurons to induce pain.^19^ These factors likely contribute to subchondral bone pain in osteoarthritis. However, blocking PGE2 and netrin-1 only partially alleviates pain. This indicates that our current understanding of the mechanisms involved in subchondral bone pain in osteoarthritis is incomplete.^9,19^

Previous studies have indicated notable increase in the regional distribution of sensory nerve endings during the development of osteoarthritis. In additional, there is a significant rise in the number of sympathetic nerve endings in the subchondral bone.^10,17,20,21^ Clinically, a close association between sympathetic nerves and joint pain has been demonstrated. β-adrenergic blockers, such as propranolol, have been shown to effectively reduce the incidence of temporomandibular joint disorder-related pain.^22–24^ Nevertheless, the way how regional sympathetic innervation in subchondral bone contributes to osteoarthritis-related pain remains elusive. Therefore, further investigation into the mechanisms through which the sympathetic nervous system regulates osteoarthritic pain is warranted.

Temporomandibular joint osteoarthritis (TMJ-OA) is a common subtype of osteoarthritis with a high incidence rate. Increase in the propensity of sympathetic nerves in the subchondral bone of the condyle has been reported in a unilateral anterior crossbite (UAC) animal model of osteoarthritis.^20^ In the present study, the UAC appliance was used to establish a TMJ-OA pain model. The hypothesis tested was that sympathetic nerves play a regulatory role in TMJ-OA related pain. Improved understanding of TMJ-OA related pain lays the foundation for developing strategies for early control of this type of debilitating pain.

## Results

### Changes in regional sympathetic nerve distribution and pain level in TMJ-OA mice

**Fig. 1:**
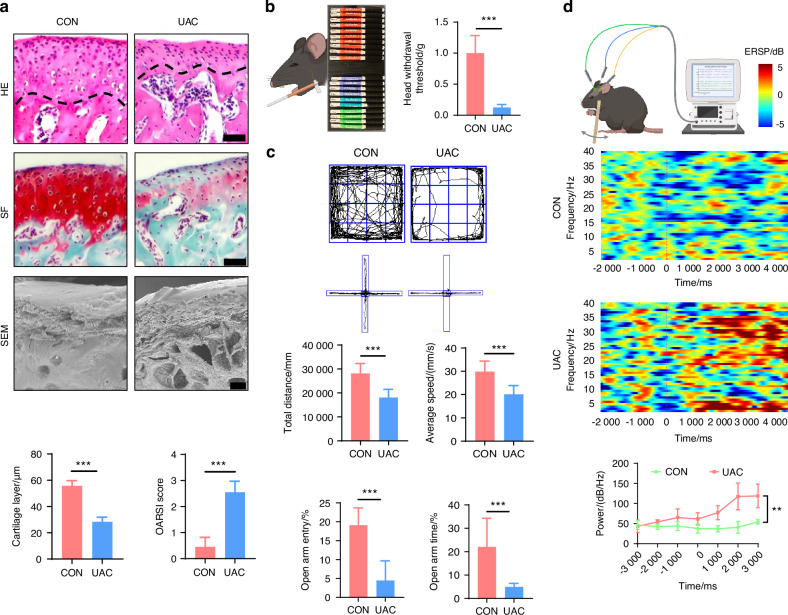
The pathological manifestations and pain behaviors of TMJ-OA mice. **a** Representative image of H&E staining (scale bars, 50 μm), SF staining (scale bars, 50 μm), and SEM (scale bars, 100 μm) of the condyles in the control (CON) and unilateral anterior crossbite (UAC) induced osteoarthritis groups with the corresponding quantification of cartilage layer thickness and OARSI scores (*n* = 3). The dashed line represents the boundary between the condylar cartilage and the subchondral bone. **b** Schematic and quantitative analysis of von-frey test (*n* = 6). **c** Representative results and quantitative analysis of the open field test and the elevated plus maze test (*n* = 6). **d** Schematic, representative results and quantitative analysis of the intracranial electroencephalogram experiment (*n* = 6). **P < 0.01, ***P < 0.001, and ****P < 0.000 1 by Student’s *t* tests. Schematic generated with Bio Render

TMJ-OA was induced using the UAC method.^25–27^ Three weeks after UAC induction, histological analyses including hematoxylin-eosin (HE) staining, safranin o-fast green (SF) staining and scanning electron microscopy (SEM) observations revealed notable osteoarthritic features in the mice of the UAC group compared to the control (CON) group (Fig. 1a). Specifically, there was a reduction in cartilage thickness, an increase in microfractures and fissures at the cartilage-bone interface, and the presence of resorption pits in the subchondral bone. Consistent with these structural changes, the Osteoarthritis Research Society International (OARSI) scores were significantly higher in the UAC-induced mice compared to the CON group. Behavioral tests are frequently employed to delineate the pain status of mice with osteoarthritis. Accordingly, we utilized the von-frey test to assess the pain sensitivity in mice, which demonstrated a decreased pain threshold in the UAC group compared to the CON group (Fig. 1b), indicative of exacerbated pain. Research has evidenced that mice in chronic pain, besides exhibiting pain-related behaviors, also display signs of depression.^28^ Consequently, we conducted two behavioral tests associated with depression: the elevated plus maze test and the open field test. The results showed that, relative to the CON group, the mice of UAC group made fewer entries into the open arms, spent less time there, and primarily confined their movements in the open field to the periphery, with a reduction in total distance traveled and average velocity, manifesting depressive-like behaviors (Fig. 1c).

**Fig. 2:**
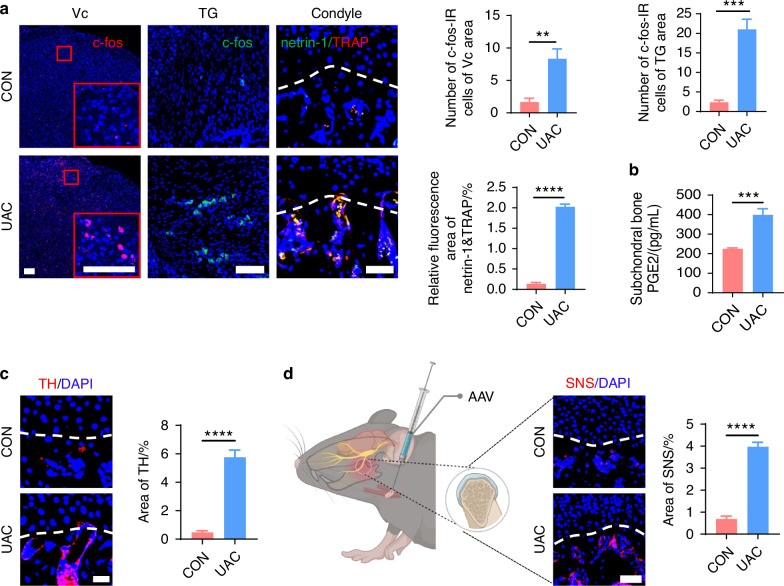
Sympathetic nerve distribution in the subchondral bone and pain activation of TMJ-OA mice. **a** Representative immunofluorescent staining imaged and quantitative analysis of DAPI (blue) and c-fos (red) of trigeminal subnucleus caudalis (Vc) (scale bars, 100 μm), DAPI (blue) and c-fos (green) of trigeminal ganglion (TG) (scale bars, 100 μm), DAPI (blue), netrin-1 (green) and TRAP (red) of the murine condyles (scale bars, 50 μm; *n* = 3). **b** ELISA of PGE2 levels in the murine subchondral bone (*n* = 3). **c** Representative immunofluorescence images and quantitative analysis of DAPI (blue), TH (red) of the murine condyles (scale bars, 25 μm; *n* = 3). **d** Schematic and representative results of viral anterograde tracing in the superior cervical sympathetic ganglion (red). Nucleus were stained with DAPI (blue). SNS means sympathetic nervous system. AAV means adeno-associated virus (scale bars, 50 μm; *n* = 3). **P < 0.01, ***P < 0.001, and ****P < 0.000 1 by Student’s *t* tests. Schematic generated with Bio Render

Studies have revealed that pain in the TMJ is hierarchically modulated by the primary somatosensory barrel field (S1BF) cortex,^29^ the caudal part of the trigeminal subnucleus caudalis (Vc), and the trigeminal ganglion (TG).^30^ Consequently, we employed electroencephalography (EEG) to examine cortical electrophysiological activity in the S1BF region and utilized immunofluorescence staining to quantify the levels of c-fos protein, a marker of neuronal activation,^31–33^ in Vc and TG. The EEG results demonstrated that, compared to the CON group, power spectral energy across all frequency bands were elevated in the S1BF cortex of the UAC group (Fig. 1d). Immunofluorescence findings further indicated a significant increase in c-fos expression in both the Vc and TG of the mice of UAC group relative to the CON group (Fig. 2a). Collectively, these outcomes illustrate that the neural activity in the various levels of pain-modulating centers is significantly enhanced in the mice of UAC group, reflecting an exacerbation of the pain phenotype. Moreover, previous studies have established a close association between locally elevated levels of osteoclast-derived netrin-1 and PGE2 with bone-originated pain.^34–37^ Consequently, we employed immunofluorescence staining and enzyme-linked immunosorbent assay (ELISA) assays to measure the contents of netrin-1 and PGE2 in the subchondral bone. Our findings revealed that compared to the CON group, the mice of UAC group showed a significant increase in netrin-1 content in the subchondral bone, which prominently co-localized with the osteoclast marker tartrate-resistant acid phosphatas (TRAP) (Fig. 2a). Additionally, the concentration of PGE2 was also significantly increased (Fig. 2b), suggesting an exacerbation of pain in the mice of UAC group.

In addition to pain manifestations, we assessed the sympathetic nerve innervation in the subchondral bone of mice through tyrosine hydroxylase (TH) immunofluorescence staining (Fig. 2c) and viral anterograde tracing (Fig. 2d). The results demonstrated a marked increase in sympathetic nerve terminals in the subchondral bone of osteoarthritic mice, with the newly added terminals predominantly clustered near the bone marrow cavity and extending toward the cartilage layer, even approaching or penetrating the tidemark demarcating the bone-cartilage boundary. Notably, however, ELISA results showed no statistically significant difference in serum norepinephrine (NE) (Fig. S1a) and PGE2 (Fig. S1b) concentrations between the UAC and CON groups. These findings indicate that UAC induction augments regional sympathetic nerve innervation and increases local PGE2 content in the subchondral bone of osteoarthritic mice, without altering their systemic activity.

Collectively, these results suggest elevated sympathetic nerves in the subchondral bone area is concurrent with augmented pain expression in TMJ-OA mice. However, whether the sympathetic nervous system is associated with the pain experienced by the mice needs to be further explored.

### Effect of sympathetic nerves on TMJ-OA pain

**Fig. 3:**
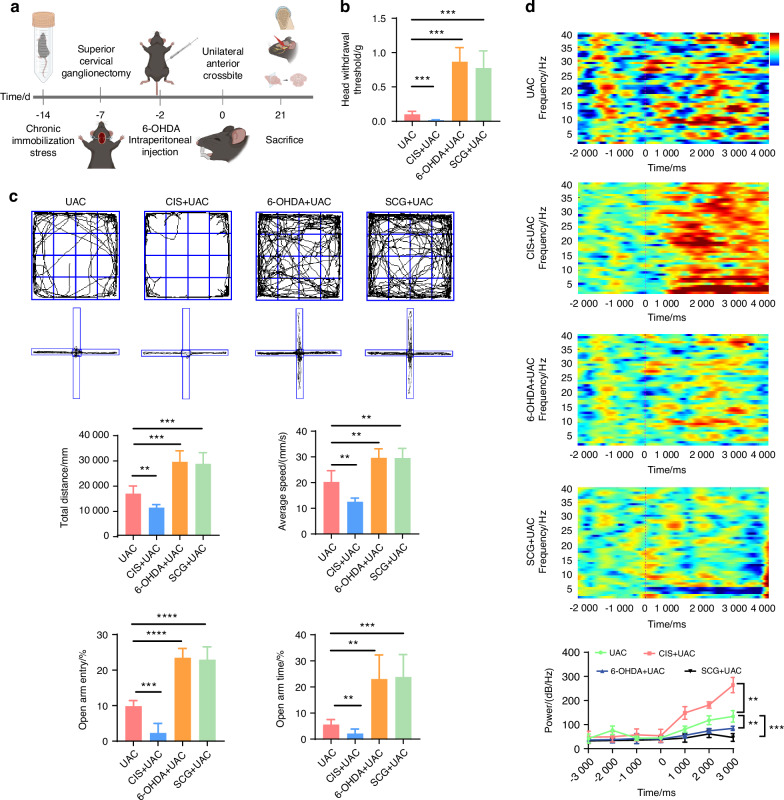
The sympathetic nervous system has a regulatory effect on the pain behaviors of TMJ-OA mice. **a** Schematic of experiment procedures. **b** Von-frey test results of mice in different groups (*n* = 6). **c** Representative results and quantitative analysis of the open field test and the elevated plus maze test (*n* = 6). **d** Representative results and quantitative analysis of the intracranial EEG experiment (*n* = 6). **P < 0.01, ***P < 0.001, and ****P < 0.000 1 by one-way ANOVA. Schematic generated with Bio Render

To elucidate the association between the sympathetic nervous system and pain modulation, we built upon the UAC model by establishing the chronic immobilization stress (CIS) model to activate sympathetic nerves, and employed superior cervical ganglionectomy (SCG),^38^ as well as intraperitoneal injections of 6-hydroxydopamine (6-OHDA),^27^ to downregulate sympathetic signaling activity in UAC mice (Fig. 3a), followed by assessing the degree of pain in mice across these different models. For SCG-treated mice, presence of ptosis (Horner’s syndrome) after ganglionectomy was indicative of successful removal of the sympathetic nerves (Fig. S2). For 6-OHDA-treated mice, bilateral ptosis observed within 6 h after the first injection was indicative of successful suppression of sympathetic signals. Immunofluorescence analysis revealed that, those subjected to CIS + UAC treatment displayed a significant increase in sympathetic nerve density in the condylar subchondral bone compared to mice in the UAC group, while those subjected to SCG + UAC and 6-OHDA + UAC showed a substantial decrease in sympathetic innervation in the condylar subchondral bone (Fig. S3). These findings were indicative of the successful establishment of a murine model for sympathetic nerve activation and blockade.

**Fig. 4:**
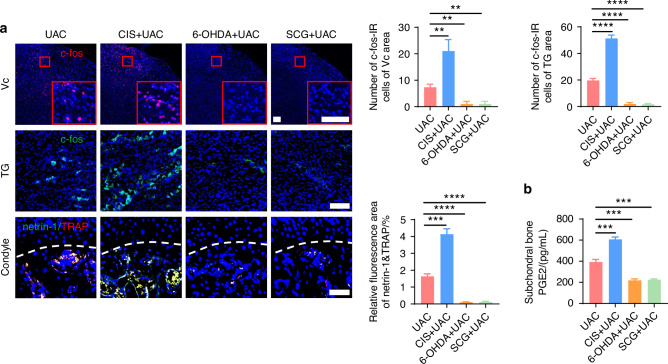
Sympathetic nerves play a regulatory role in TMJ-OA pain. **a** Representative immunofluorescent staining images and quantitative analysis of DAPI (blue) and c-fos (red) of the trigeminal subnucleus caudalis (Vc) (scale bars, 100 μm), DAPI (blue) and c-fos (green) of trigeminal ganglion (TG) (scale bars, 100 μm), DAPI (blue), TRAP (red) and netrin-1 (green) of the murine condyles (scale bars, 50 μm; *n* = 3). **b** ELISA of subchondral bone PGE2 levels in mice (*n* = 3). **P < 0.01, ***P < 0.001, and ****P < 0.000 1 by one-way ANOVA

Subsequently, we examined the pain conditions of mice across different groups. Von-Frey tests indicated that, compared to the mice of UAC group, the mice of CIS + UAC group had a significantly lower pain threshold, whereas the mice of 6-OHDA + UAC and SCG + UAC groups showed a marked increase in pain threshold (Fig. 3b). Results from the open field and elevated plus maze tests (Fig. 3c) demonstrated that, compared to the mice of UAC group, mice of CIS + UAC group had a significantly reduced total movement distance and average velocity in the open field, concentrating their activities at the edges. In the elevated plus maze, they showed a decreased frequency of entries into open arms and shorter stay times, exhibiting evident depressive-like behavior. Conversely, the mice of 6-OHDA + UAC and SCG + UAC groups displayed a significant alleviation of depression. Electroencephalogram (EEG) results showed that, compared to the mice of UAC group, the mice of CIS + UAC group had significantly elevated electrophysiological signals in the S1BF cortex, whereas the mice of 6-OHDA + UAC and SCG + UAC groups exhibited reduced cortical signals (Fig. 3d). Immunofluorescence staining results (Fig. 4a) revealed that, in comparison to the mice of UAC group, the mice of CIS + UAC group had increased c-fos expression in both the Vc and TG, indicating enhanced neuronal activity, whereas the mice of 6-OHDA + UAC and SCG + UAC groups showed reduced c-fos expression in the Vc and TG, pointing to decreased neuronal activity. Moreover, compared to the mice of UAC group, the mice of CIS + UAC group had higher levels of pain-related substances including netrin-1 and PGE2 in the subchondral bone, whereas the mice of 6-OHDA + UAC and SCG + UAC groups had lower levels of netrin-1 (Fig. 4a) and PGE2 (Fig. 4b) in their subchondral bone. These findings collectively suggest that pain is exacerbated when the sympathetic nervous system is activated and relieved when sympathetic signal is inhibited.

Collectively, these findings indicate that the sympathetic nervous system plays a regulatory role in osteoarthritic pain. However, the specific mechanisms by which the sympathetic nervous system regulates osteoarthritic pain remain unclear and require further investigation.

### Spatiotemporal relation between sympathetic and sensory nerves in TMJ-OA

Research indicates that TMJ-OA pain largely originates from an increase in sensory nerve terminals and their sensitization within the subchondral bone of the joint.^8^ Thus, the regulation process of sympathetic nerves on osteoarthritic pain raises our interest in whether it is related to sensory nerves. To explore this, we employed immunofluorescence staining (Fig. S4) and viral anterograde tracing techniques (Fig. S5) to investigate the temporal and spatial relationship between sympathetic and sensory nerves during the course of osteoarthritis development.

**Fig. 5:**
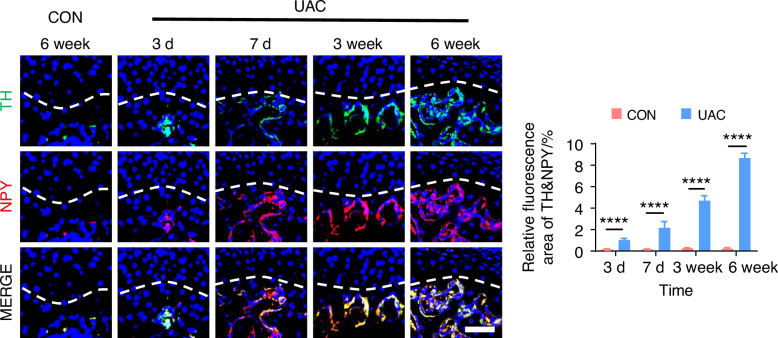
Spatiotemporal distribution of sympathetic nerves during TMJ-OA progression. Representative immunofluorescent staining images and quantitative analysis of DAPI (blue), TH (green), NPY (red) of the murine condyles (scale bars, 50 μm; *n* = 3). ****P < 0.000 1 by Student’s *t* tests

**Fig. 6:**
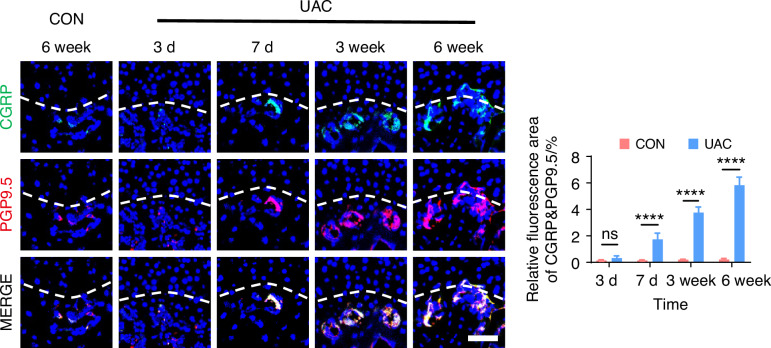
Spatiotemporal distribution of sensory nerves during TMJ-OA progression. Representative immunofluorescent staining images and quantitative analysis of DAPI (blue), CGRP (green) and PGP9.5 (red) of the murine condyles (scale bars, 50 μm; *n* = 3). ns means not significant. ****P < 0.000 1 by Student’s *t* tests

By using immunofluorescence staining, we observed the innervation of sympathetic (Fig. 5 and Fig. S6) and sensory (Fig. 6 and Fig. S7) nerves in the subchondral bone of early TMJ-OA. Among these, TH and NPY served as markers for sympathetic nerves, while CGRP and PGP 9.5 were markers for sensory nerves. The results showed that in the mice of CON group, the content of sympathetic and sensory nerve terminals in the subchondral bone remained consistently low and did not exhibit a significant increase over time. In contrast, in mice of UAC group, just 3 days after TMJ-OA induction, the distribution of sympathetic nerve terminals in subchondral bone exceeded that of the mice of CON group, while the sensory nerve terminals in the subchondral bone were significantly more abundant on day 7 post-UAC induction compared to those in the mice of UAC group. As TMJ-OA progressed, sympathetic and sensory nerve innervation persistently intensified, demonstrating a tendency to progressively extend towards the interface between bone and cartilage, even breaching the tidemark. This finding implies that, during the development of TMJ-OA, the increase in sympathetic nerve innervation in the subchondral bone precedes that of sensory nerves. From a temporal standpoint, the results suggested that sympathetic nerves may exert a modulatory influence on sensory nerves. Of particular note, the close distribution of sympathetic and sensory nerves suggests a possible direct interaction between them.

**Fig. 7:**
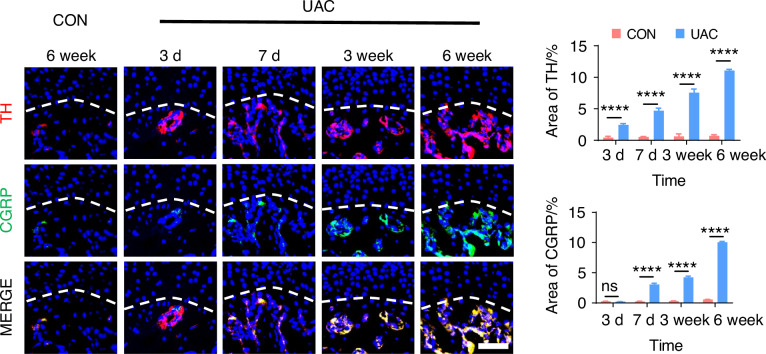
The immunofluorescence staining results showing the distribution relationship between sympathetic and sensory nerves during the progression of TMJ-OA. Representative immunofluorescent staining images and quantitative analysis of DAPI (blue), TH (red), and CGRP (green) of the murine condyles (scale bars, 50 μm, *n* = 3). ns means not significant. ****P < 0.000 1 by Student’s *t* tests

**Fig. 8:**
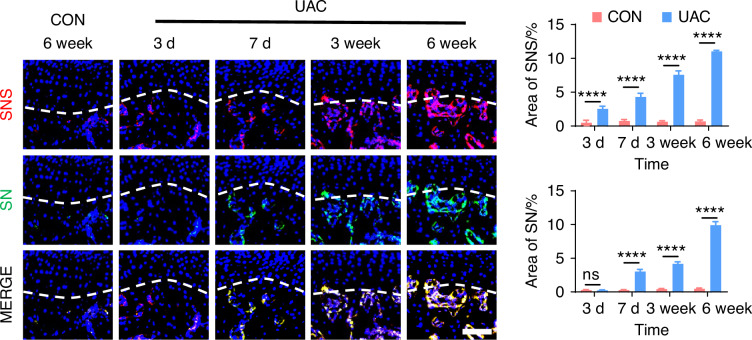
The viral anterograde tracing results demonstrating the distribution relationship between sympathetic and sensory nerves during the progression of TMJ-OA. Representative results, and quantitative analysis of viral anterograde tracing in the trigeminal ganglion (green) and the superior cervical sympathetic ganglion (red). Nucleus were stained with DAPI (blue). SNS means sympathetic nervous system. SN means sensory nerves (scale bars, 50 μm, *n* = 3). ns means not significant. ****P < 0.000 1 by Student’s *t* tests

To delve deeper into the possibility of sympathetic regulation of sensory nerves, we employed immunofluorescence staining (Fig. 7 and Fig. S8) and viral anterograde tracing (Fig. 8 and Fig. S9) to examine the spatial relationship between these two neural systems. Immunofluorescence staining results revealed that, compared to the mice of CON group, the sympathetic and sensory nerves in the mice of UAC group were closely intertwined. With the progression of osteoarthritis, innervation by both types of nerves significantly increased, extending progressively towards the cartilage-bone interface and even penetrating the tidemark. The viral anterograde tracing confirmed this trend, indicating a high degree of congruency in their spatial positioning. These findings provide compelling morphological evidence of a tightly correlated spatial arrangement between the two neural systems, which paves the way for further exploration into their mutual interactions and potential regulatory mechanisms in disease progression. In addition, we also applied the Von-Frey (Fig. S10a, b), open field and elevated plus maze (Fig. S10c) tests at different time points after UAC induction. The results showed that pain-related behaviors appeared in the mice 3 days after UAC induction and progressively worsened. This time point coincides with the increase in immunofluorescent signal of sympathetic nerves, further corroborating the association between sympathetic nerves and TMJ-OA pain.

These findings suggest that, during the progression of TMJ-OA, sympathetic nerves infiltrate the subchondral bone earlier than sensory nerves and run in close proximity to them. Based on their distribution patterns and sequential growth over time, we speculate that, in the course of TMJ-OA, sympathetic nerves might modulate the pain by regulating the growth of sensory nerve terminals in the subchondral bone, thereby exerting control over TMJ-OA pain.

### Influence of regional sympathetic nerves on sensory nerves in TMJ-OA

**Fig. 9:**
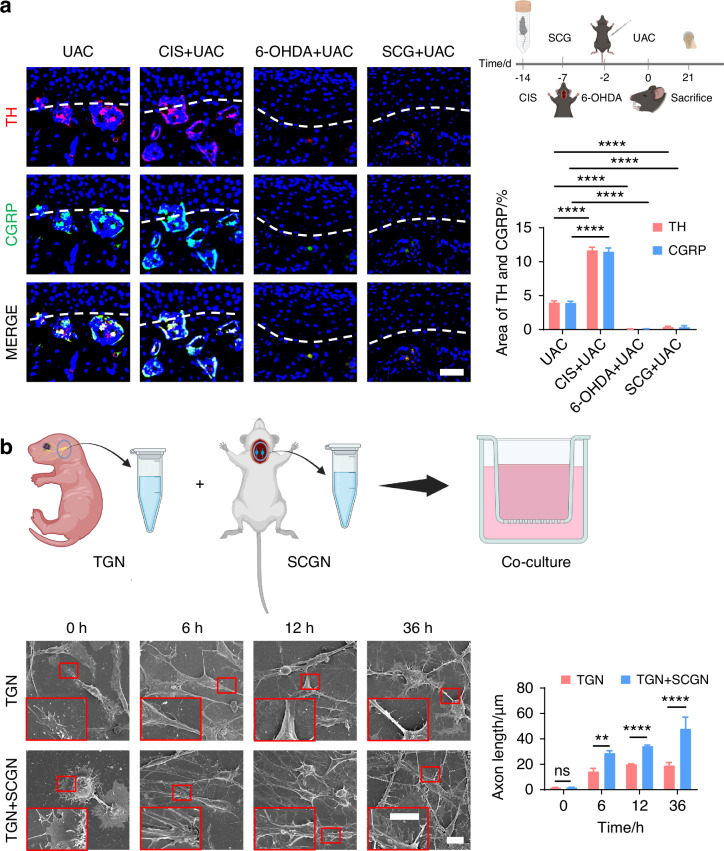
The regulatory effect of sympathetic nerves on sensory nerves. **a** Schematic, representative immunofluorescent staining images and quantitative analysis of DAPI (blue), TH (red) and CGRP (green) of the murine condyles 3 weeks after the UAC induction (scale bars, 50 μm; *n* = 3). **** means *P* < 0.0001 by one-way ANOVA. **b** Schematic of the experiment procedures and representative images of SEM of the TG neurons (TGN) group and TGN + SCG neurons (SCGN) group with the corresponding statistical results (scale bars, 10 μm; *n* = 3). ns means not significant, **P < 0.01, and ****P < 0.000 1 by Student’s *t* tests. Schematic generated with Bio Render

**Fig. 10:**
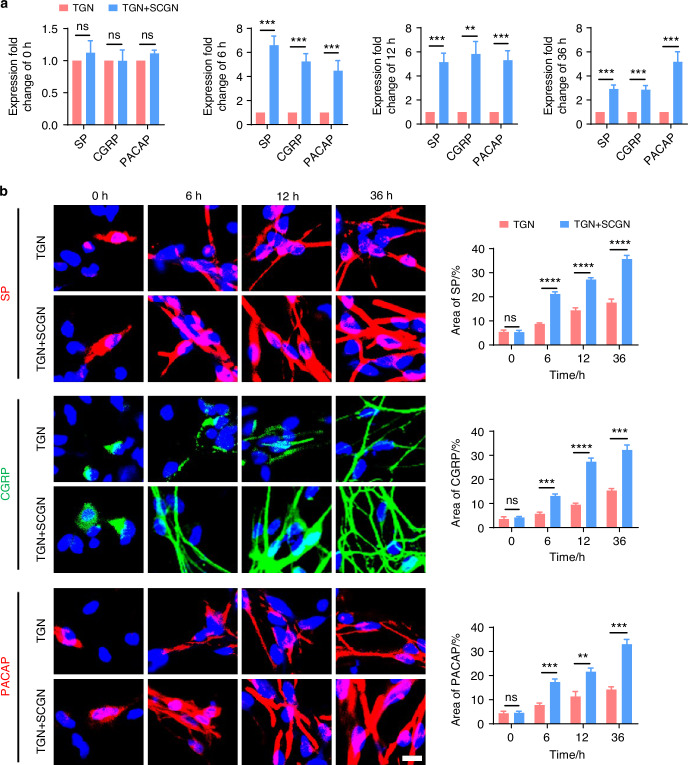
Sympathetic nerves promote sensory innervation. **a** qRT-PCR analysis of the gene expression of substance P (SP), calcitonin gene-related peptide (CGRP) and pituitary adenylate cyclase-activating polypeptide (PACAP) in the TGN group and TGN + SCGN group in different time points (*n* = 3). **b** Representative immunofluorescent staining images and the quantitative analysis of the expression of DAPI (blue), SP (red), CGRP (green) and PACAP (red) in the TGN group and TGN + SCGN group in different time points (scale bars, 10 μm; *n* = 3). ns means not significant, **P < 0.01, ***P < 0.001, and ****P < 0.000 1 by Student’s *t* tests

To further clarify whether the sympathetic nervous system modulates TMJ-OA pain via regulation of sensory nerves, we employed immunofluorescence staining to analyze the distribution of sensory nerves in the subchondral bone in mice under sympathetic nerve activation and blockade conditions. Three weeks after UAC induction, compared to the mice of UAC group, in the mice of CIS + UAC group where sympathetic nerves were activated, sensory nerves closely accompanied sympathetic nerves, and the number of sensory nerve terminals significantly increased alongside the elevation of sympathetic nerves. Conversely, in the mice of 6-OHDA + UAC and SCG + UAC groups, where sympathetic nerves were blocked, the sensory nerves decreased along with the decline in sympathetic nerves (Fig. 9a). We then established a co-culture system of trigeminal ganglion neurons (TGN) and superior cervical ganglion neurons (SCGN) to investigate the direct regulatory effect of sympathetic neurons on sensory neurons in vitro. Scanning electron microscopy (Fig. 9b) and β3-tubulin immunofluorescence staining (Fig. S11) were employed to observe the morphology and axonal extension of TGNs. The results showed that, starting from 6 h of co-culture, TGNs grown together with sympathetic neurons displayed a markedly accelerated increase in axon elongation and expansion compared to those cultured alone. This facilitative effect became increasingly pronounced as the culture duration extended. In addition, we also performed the quantitative real-time polymerase chain teaction (qRT-PCR) as well as immunofluorescence staining of CGRP, substance P (SP) and pituitary adenylate cyclase activating peptide (PACAP) at different time points. The results show that the gene (Fig. 10a) and protein expression levels (Fig. 10b) of these three substances were significantly higher in TGN + SCGN co-culture group compared to the TGN group at 6, 12 and 36 h.

These findings confirm a regulatory influence of sympathetic nerves on sensory nerves. However, the precise mechanisms by which sympathetic nerves regulate sensory nerves remain unclear and warrant further investigation.

### How sympathetic nerves regulate sensory nerves

**Fig. 11:**
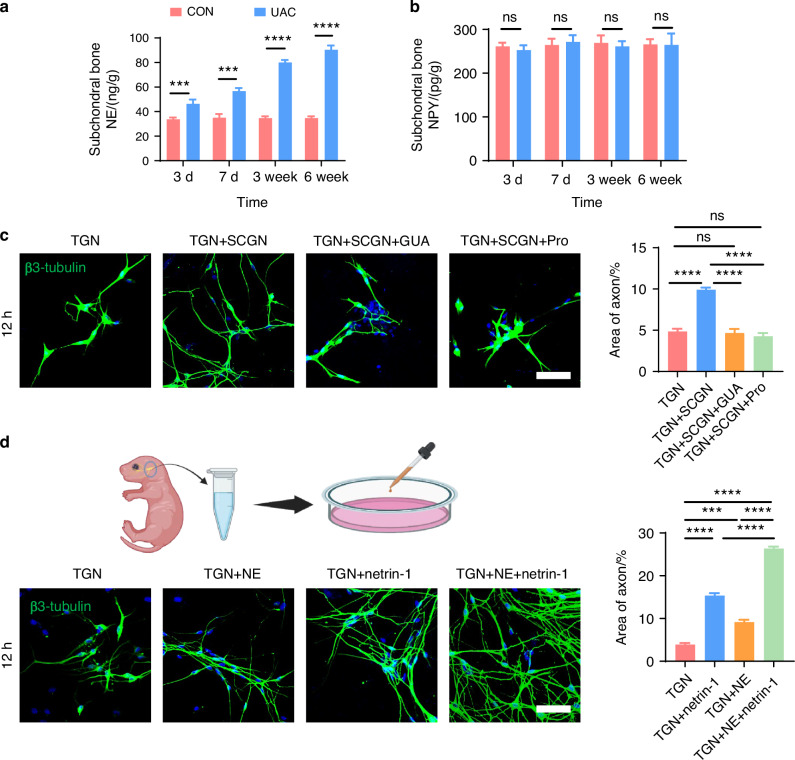
Sympathetic nerves regulate sensory nerves axon growth through the release of NE. **a** ELISA of subchondral bone NE levels in mice (*n* = 3). ***P < 0.001, and ****P < 0.000 1 by Student’s *t* tests. **b** ELISA of subchondral bone NPY levels in mice (*n* = 3). ns means not significant by Student’s *t* tests. **c** Representative immunofluorescent staining images and quantitative analysis of DAPI (blue) and β3-tubulin (green) of the TGN group, TGN + SCGN group, TGN + SCGN + guanethidine (GUA) group, and TGN + SCGN + Propranolol (Pro) group (scale bars, 60 μm; *n* = 3). **d** Schematic, representative immunofluorescent staining results and quantitative analysis of DAPI (blue) and β3-tubulin (green) of the TGN group, TGN + NE group, TGN + netrin-1 group and TGN + NE + netrin-1 group (scale bars, 60 μm; *n* = 3). **c**, **d** ns means not significant, ***P < 0.001, and ****P < 0.000 1 by one-way ANOVA. Schematic generated with Bio Render

Previous studies indicated that sensory nerves are modulated by sympathetic nerves through neurotransmitter release.^39,40^ Among the neurotransmitters, norepinephrine (NE) and neuropeptide Y (NPY) are particularly implicated in pain regulation.^41–45^ Accordingly, ELISA was used to measure NE and NPY levels in the subchondral bone. A significant increase in NE levels was found in the UAC group, compared to the CON group (Fig. 11a). No significant difference was noted for NPY between the two groups (Fig. 11b). This finding suggests that NE may play a role in the crosstalk between sympathetic and sensory nerves in TMJ-OA.

**Fig. 12:**
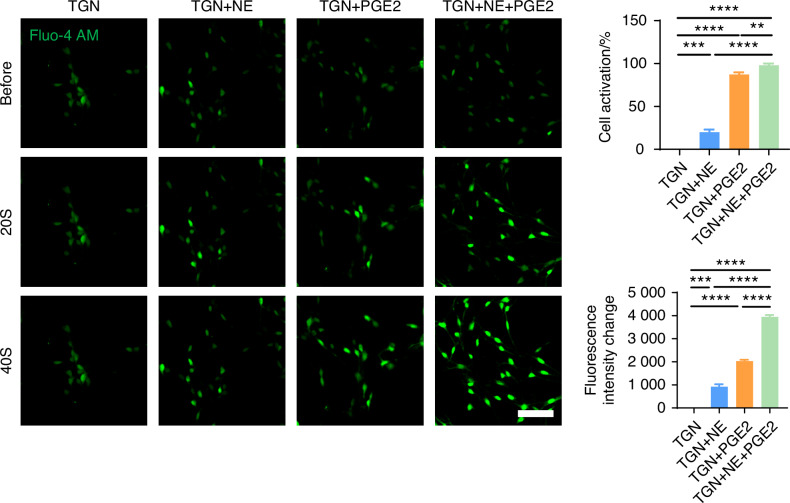
NE released by sympathetic nerves can modulate the activation of sensory nerves. Representative results and quantitative analysis of live-cell calcium imaging (scale bars, 60 μm; *n* = 3). **P < 0.01, ***P < 0.001, and ****P < 0.000 1 by one-way ANOVA

Guanethidine (GUA) is commonly used to block NE release from sympathetic nerves.^46–48^ Propranolol (Pro), a non-selective β-receptor antagonist, is frequently employed to inhibit β-receptors.^49,50^ To elucidate the regulatory effect of NE on sensory neurons, we introduced guanethidine (GUA) and propranolol (Pro) into the TGN + SCGN co-culture system in vitro, with the aim of inhibiting NE secretion and blocking β-receptors on the sensory neurons, respectively. Under equal cultivation periods, our experimental results showed that, compared to the TGN group, the TGN + SCGN group exhibited a significantly greater axonal expansion area of sensory neurons. However, axonal growth of sensory neurons was notably reduced when added with NE blockers in the groups of GUA (TGN + SCGN + GUA) or Pro (TGN + SCGN+Pro), similar to the growth state observed in the TGN group (Fig. 11c). This finding robustly demonstrates that suppressing NE production or its engagement with β-receptors on sensory neurons can effectively reverse the axon growth advantage induced by co-culture. Consequently, this confirms that sympathetic neurons activate sensory neurons through NE release, playing a crucial role in facilitating axonal elongation. To further validate the regulatory role of NE on sensory neurons, we directly treated TGNs with NE and employed netrin-1 and PGE2, substances previously shown to modulate sensory neurons,^34,36,37^ as positive controls and the objective was to observe the effect of NE on sensory neuron regulation. Our results demonstrated that NE significantly promoted axonal growth of sensory neurons (Fig. 11d). Furthermore, calcium imaging revealed that NE notably activated these neurons (Fig. 12 and Video. S1–4), leading to heightened intracellular calcium signal and obvious calcium oscillations activity after adding NE.^51^ Additionally, we uncovered that when NE acts in conjunction with netrin-1 or PGE2 on sensory neurons, it synergistically amplifies the effects of netrin-1 or PGE2 on these neurons (Figs. 11d, 12 and Video. S1–4). Collectively, these findings indicate that NE can directly or cooperatively with netrin-1 and PGE2 facilitate the growth and activation of sensory neurons, contributing to aberrant pain sensations.

### The effect of norepinephrine on TMJ-OA pain

**Fig. 13:**
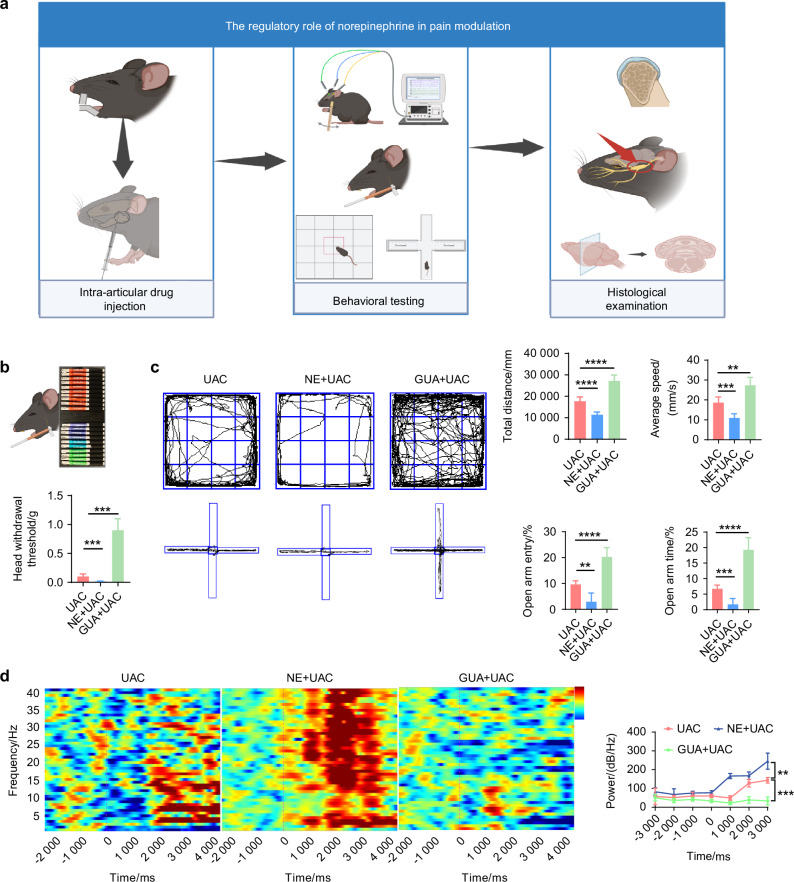
Norepinephrine has a regulatory effect on the pain behaviors of TMJ-OA mice. **a** Schematic of the experiment procedures. **b** Schematic and quantitative analysis of von-frey test (*n* = 6). **c** Representative results and quantitative analysis of the open field test and the elevated plus maze test (*n* = 6). **d** Representative results and quantitative analysis of the intracranial EEG experiment (*n* = 6). **P < 0.01, ***P < 0.001, and ****P < 0.000 1 by one-way ANOVA. Schematic generated with Bio Render

To verify if sympathetic nerves regulate pain in TMJ-OA via NE secretion, intra-articular injections of guanethidine and NE were administered to mice in the UAC group. Changes in pain status were observed compared to mice with TMJ-OA that were injected with sterile physiological saline (Fig. 13a).

**Fig. 14:**
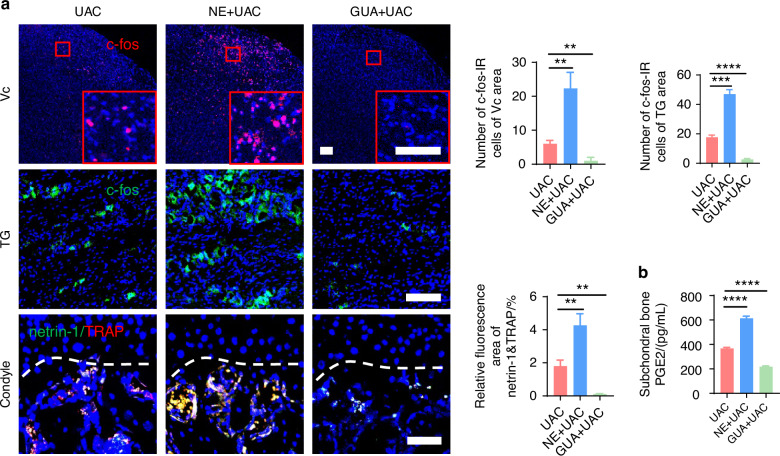
Norepinephrine plays a regulatory role in TMJ-OA pain. **a** Representative immunofluorescent staining images and quantitative analysis of DAPI (blue) and c-fos (red) of trigeminal subnucleus caudalis (Vc) (scale bars, 100 μm), DAPI (blue) and c-fos (green) of trigeminal ganglion (TG) (scale bars, 100 μm), DAPI (blue), TRAP (red) and netrin-1 (green) of the murine condyles (scale bars, 50 μm; *n* = 3). **b** ELISA of murine subchondral bone PGE2 levels (*n* = 3). **P < 0.01, ***P < 0.001, and ****P < 0.0001 by one-way ANOVA

The von-frey results indicated that, compared to the mice of UAC group, the pain threshold was significantly reduced in the mice of NE + UAC group, whereas it was notably elevated in the mice of GUA + UAC group (Fig. 13b). In the open field test and elevated plus maze assessment (Fig. 13c), the mice of NE + UAC group manifested clear depressive-like behaviors. Conversely, the mice of GUA + UAC group displayed exploratory behaviors and a marked reduction in depressive symptoms. Electrophysiological analyses revealed that, compared to the mice of UAC group, the mice of NE + UAC group showed significantly heightened cortical electrical activity in the S1BF region, whereas the mice of GUA + UAC group exhibited a decrease in such signals (Fig. 13d). Immunofluorescence staining results (Fig. 14a) demonstrated that, compared to the mice of UAC group, the mice of NE + UAC group exhibited increased c-fos expression in the Vc and TG regions, suggesting enhanced neuronal activity. Conversely, the mice of GUA + UAC group showed reduced c-fos expression in the Vc and TG, indicating lowered neural activity. Moreover, in comparison to the mice of UAC group, the mice of NE + UAC group had higher levels of pain-related substances netrin-1 and PGE2 in their subchondral bone, whereas the mice of GUA + UAC group displayed lower concentrations of netrin-1 (Fig. 14a) and PGE2 (Fig. 14b) in the subchondral bone. These findings collectively imply that a local increase in NE exacerbates pain, whereas the blockade of NE alleviates pain symptoms.

These findings indicate that sympathetic nerves have a regulatory effect on TMJ-OA pain, and this regulation is mediated through the release of NE by sympathetic nerves, which acts on sensory nerves to promote the growth and activation of sensory nerve terminals, thereby modulating TMJ-OA pain.

## Discussion

TMJ-OA induced chronic pain is highly prevalent and significantly impacts the physical and mental well-being of those affected by the disease. However, the mechanisms underlying its occurrence remain poorly understood. The present study unveiled the involvement of sympathetic nerves in joint pain regulation. A UAC model was used to induce TMJ-OA in mice. Chronic immobilization stress, 6-OHDA injections, and superior cervical ganglionectomy were used to regulate sympathetic nerves. Histological analysis of joint specimens after the operations identified the spatiotemporal relationship between sympathetic and sensory nerves in early-stage TMJ-OA. Immunofluorescence analyses and behavioral characterizations showed that sympathetic nerves infiltrate the subchondral bone earlier than sensory nerves, and play a regulatory role in TMJ-OA pain. Activation of sympathetic nerves by CIS facilitated sensory nerve growth and aggravated pain-related behaviors. Conversely, suppression of sympathetic nerves through 6-OHDA injections or SCG reduced these effects. These findings were further supported by the results of in vitro experiments. Taken together, the study identifies that sympathetic nerves, via the release of the neurotransmitter NE, enhance the growth of sensory nerve endings in subchondral bone, activate sensory neurons, and co-operate with local neuroactive factors netrin-1 and PGE2 in the subchondral bone to exacerbate osteoarthritis-related pain. This discovery highlights the impact of local sympathetic nerves on sensory nerve infiltration and activation through releasing NE. This provides crucial insights into the mechanisms of osteoarthritic pain, and suggests novel strategies for its therapeutic intervention (Graphic abstract).

During the early stages of TMJ-OA, the primary source of pain originates from the subchondral bone. Pathological remodeling of the subchondral bone often involves the growth of new nerves and blood vessels.^17,18^ The regional increase in sensory nerve endings is thought to contribute to TMJ-OA related pain.^52,53^ This aligns well with the findings of the present, which demonstrate a significant increase in sensory nerve endings within the subchondral bone during the progression of TMJ-OA. Netrin-1 is an axon guidance protein secreted by osteoclasts.^54^ It promotes the innervation of sensory nerves during subchondral bone remodeling in TMJ-OA.^9^ Regional increase in PGE2 levels within the remodeled subchondral bone also activates sensory nerve endings, augmenting pain levels.^19^ Nevertheless, blocking netrin-1 and PGE2 only partially relieves osteoarthritis pain. This finding suggests that additional contributory sources for osteoarthritis pain exist. Serendipitously, a notable increase in sympathetic nerves in the subchondral bone region was observed in the present work during the development of TMJ-OA. These sympathetic nerves release NE, which acts upon sensory nerve endings to activate the growth of sensory neurons. This finding offers fresh insights into the origin of TMJ-OA pain and provides potential therapeutic directions for its management.

Pain perception is a complex, multi-level regulatory process that spans from peripheral nociceptors at the site of injury to the central nervous system.^55^ The involvement of sympathetic nerves in pain regulation has long been investigated in the central nervous system. For example, the locus coeruleus in the brainstem releases NE, which ascends to the cerebral cortex and promotes chronic pain in the trigeminal system.^56,57^ At the ganglion level, sympathetic nerve sprouting in the dorsal root ganglia releases neurotransmitters that act on sensory neurons to induce pain.^42^ This study fills a gap on the mechanism of the sympathetic regulation of the sensory nerve innervation and function in osteoarthritic pain. It was uncovered that sympathetic nerves exert their influence by releasing NE, which acts on beta receptors located on sensory nerves. This action is compounded by the presence of local sensory nerve activators such as netrin-1 and PGE2, promoting the growth and activation of sensory nerves. This discovery provides a foundational basis for studying the mechanisms by which the sympathetic nervous system modulates peripheral pain.

It is prudent to point out that there was a marked increase in the density of the sympathetic nerve fibers as early as day 3 in the UAC model. This increase preceded the increased distribution of sensory nerve fibers. The phenomenon may be attributed to the ability of sympathetic nerves to rapidly sprout at their axonal terminals during inflammation or neural injury.^42,58^ Upon further exploration, it was found that when sympathetic nerves were activated in TMJ-OA mice, a subsequent increase in sensory nerve fibers in the condylar subchondral bone occurred, which was accompanied by exacerbated pain. Conversely, after sympathetic nerve blockade, the number of sensory nerve fibers in the subchondral bone decreased significantly, alleviating pain. This outcome supports the hypothesis that sympathetic nerves have a regulatory effect on pain, possibly through modulation of sensory neuron activation and axon growth. Previous studies have indicated that sympathetic nerves regulate neurons by releasing neurotransmitters such as NE.^42–45,59^ NE can activate neuronal cells by mediating an inositol 3-phosphate-dependent mechanism, leading to intracellular calcium oscillations and neuronal cell activation.^51^ Moreover, NE acts on β-receptors in glial cells to activate c-AMP-dependent protein kinase. This kinase phosphorylates cytoskeletal proteins, inducing morphological changes in the glial cells.^60,61^ Consistent with these previous findings, the present study showed that the NE content in the condylar subchondral bone significantly increased during the development of osteoarthritis in the TMJ. The results indicate that NE promotes sensory neuron activation and axon growth, thus exacerbating TMJ-OA pain. This provides evidence for the regulatory role of sympathetic nerves in TMJ-OA pain, specifically through the action of NE on sensory nerve fibers.

Two substances closely related to pain are regionally produced in large quantity during the progression of TMJ-OA. The first is netrin-1, which functions as a classic axon growth-promoting factor.^34,37^ The second is PGE2, which activates sensory nerves.^36^ These two substances were selected as positive controls to study their combined effects with NE on sensory neurons. The results showed that NE can synergize with the actions of netrin-1 and PGE2. Given that NE interacts synergistically with these two substances, it further contributes to the occurrence of pain. However, the exact molecular mechanisms underlying this synergy remain unclear.

Previous research has demonstrated that sympathetic nerves can promote subchondral bone remodeling. During this process, substantial amounts of PGE2^19^ and netrin-1^9^ are released. Sympathetic nerves may exacerbate pain by promoting subchondral bone remodeling, leading to the release of additional sensory nerve activators such as PGE2 and netrin-1. Therefore, sympathetic nerves potentially amplify pain by direct stimulation of sensory nerves, and indirect facilitation of pain-causing factors during the bone remodeling process. Other studies have suggested that inflammatory mediators and neurotrophic factors, such as nerve growth factor present in the subchondral bone, also play a role in regulating osteoarthritis pain.^4^ Moreover, the involvement of NE in modulating bone remodeling has been reported.^62^ Substances like PGE2 produced during this process also play regulatory roles over osteoarthritis-related pain.^19^ Consequently, sympathetic nerves may indirectly influence osteoarthritis pain by regulating bone remodeling. The present experimental design did not exclude the impact of factors other than sympathetic nerves on pain, or rule out the indirect effects of sympathetic nerves on pain perception. To address these gaps, subsequent experimental plans should include the employment of genetically-modified mice with reduced NE release capacity in sympathetic nerves, and those with selective knock-down of adrenergic β2 receptors, on sensory nerves. This approach should help further clarify the direct regulatory effect of sympathetic nerves on sensory nerves innervation and activation.

Although limitations are present in the study, it contributes to filling the knowledge gap concerning the specific mechanisms by which regional sympathetic nerves regulate pain in TMJ-OA. This is achieved through the release of the neurotransmitter NE, which facilitates the growth and activation of sensory nerves, either directly or synergistically with netrin-1 and PGE2. These findings shed light on a new avenue for regulating sympathetic signals for the treatment of TMJ-OA related pain.

## Material and methods

### Animal models

Sixty 8-week-old female C57BL/6J mice (17–19 g each), twenty 6-week-old adult female Sprague-Dawley (SD) rats (~300 g), as well as twenty-five newborn Sprague-Dawley rats (1–5 days) were obtained from the Experimental Animal Center of Air Force Medical University (AFMU). Animal experiments were approved by the AFMU Institutional Ethics Committee (Approval No. 2021-001). All experiments were conducted in compliance with the guidelines of “Animal Research: Reporting of In Vivo Experiments” for all animal research. C57/6 J mice were employed to establish osteoarthritis models as well as models with sympathetic nerve blockage and activation, followed by behavioral tests on the mice or tissue collection for histopathological experiments. SD rats were used to construct osteoarthritis models and subsequently to harvest superior cervical ganglion cells for co-culture experiments. Neonatal SD rats were utilized to extract trigeminal ganglion cells for in vitro cellular experiments.

The mice were randomly assigned to two groups: the UAC appliance-induced osteoarthritis (UAC) group, and the sham-operated control (CON) group (*n* = 6). The mice from the UAC group were anesthetized with intraperitoneal injection of 1% pentobarbital sodium. A UAC condition was induced in the dentition of each mouse.^63^ In the UAC group, a 1.5 mm long metal tube was bonded to the left maxillary incisor using zinc phosphate cement. In addition, a 5 mm long tube, bent at a 135° angle at one end, was bonded onto the left mandibular incisor. In the CON group, the mice underwent the same anesthesia procedures but without UAC induction.

In the osteoarthritis rat model, the UAC appliance consisted of a 3 mm long metal tube, prepared with a 25-gauge needle. The tube was glued to the left upper incisor of each rat. In addition, an 8 mm long metal tube, prepared with a 20-gauge needle and bent at one end to a 135° angle, was glued to the left lower incisor of the rat to form a guide plate.

#### Chronic immobilization stress (CIS) model^27^

Each mouse was placed in a 50 mL ventilated laboratory centrifuge tube for 4 h daily (*n* = 6). In the control group, the mice were immediately removed from the centrifuge tube after placement. In the CIS + UAC group, the mice underwent a 2-week period of CIS preconditioning prior to UAC induction. The mice were euthanized 3 weeks after UAC induction.

#### Chemical sympathectomy

6-hydroxydopamine (6-OHDA, H4381, MilliporeSigma, Burlington, MA, USA) was dissolved in sterile physiological saline containing 0.02 mg/mL L-ascorbic acid (A8100, Solarbio, Life Sciences, Beijing, China). On the fourth and 3rd days prior to UAC induction, the mice were received with 100 mg/kg and 250 mg/kg of intraperitoneal injection of 6-OHDA, respectively (*n* = 6). The control group received an equivalent volume of sterile physiological saline containing 0.02 mg/mL L-ascorbic acid instead of 6-OHDA (*n* = 6). The mice were euthanized 3 weeks after UAC induction.

#### Superior cervical ganglionectomy (SCG)

In the surgical group, the mice were anesthetized with intraperitoneal injection of 1% pentobarbital sodium. The surgical procedure involved exposing the bifurcations of both common carotid arteries, locating the superior cervical ganglia situated posterior to these bifurcations, and then removing the superior cervical ganglia using forceps (*n* = 6). In the control group, the bifurcations of the common carotid arteries were exposed without excision of the superior cervical ganglia (*n* = 6).

After SCG surgery, the mice exhibited signs of Horner syndrome, including ptosis of the eyelid. Unilateral anterior crossbite induction was performed on those mice 1 week after surgical recovery. The mice were euthanized 3 weeks after UAC induction.

### Joint injection

Each mouse was anesthetized with a mixture of 2% isoflurane and oxygen, and then deeply anesthetized with pentobarbital. A Hamilton microinjector syringe needle was used to pierce the zygomatic arch between the eyes and ears, sliding along the bone wall to reach the TMJ. Three weeks after UAC induction, norepinephrine (NE, GC30744, 1 mg/mL, dissolved in sterile saline, GLPBIO Technology LLC, Montclair, CA, USA) was injected into the joint cavity at a dose of 10 μL (*n* = 6). Behavioral and histological assessments were conducted 6 h after injection. Three weeks after UAC induction, guanethidine (30 mg/kg, GC66202, dissolved in sterile saline, GLPBIO, USA) was administered via intra-articular injection at a volume of 10 μL per day for three consecutive days (*n* = 6). Behavioral and histological evaluations were performed on the 4th day. In the control group, the mice received equivalent volumes of sterile saline injection (*n* = 6).

### Electroencephalography (EEG)

After the mice were anesthetized, a 2-cm incision was made from the head to the neck to expose the skull. This was followed by precise stereotaxic implantation of a single insulated stainless-steel electrode with a diameter of 200 μm (Jingong Cat# QZY2, Wenzhou, China) into the primary somatosensory barrel field (S1BF) at coordinates X = ± 2.6, Y = −1.6, Z = −1.23. A reference electrode was placed within the cerebellum at coordinates X = 0, Y = −5.5, Z = −2.5. The electrodes were secured to the skull using dental cement (SuperBond C&B, SUN Medical, Moriyama, Japan). Following surgery, the mice were gently placed on a heating pad until they regained consciousness and motor activity.

Testing was performed after a recovery period of 7 days. The mandibular and facial regions of different groups of mice (*n* = 6) were stimulated ten times using a fine brush. Each stimulation lasted for 5 s, with an interval of 30 s between stimulations. When a force of 0.5 N was applied to the mandibular and facial area for 5 s, the EEG activity in the S1BF was amplified, filtered (high-pass: 0.2 Hz; low-pass: 40 Hz), and recorded using an EEG monitoring system (SOLAR3000N, Beijing, China) at a sampling rate of 100 Hz.^18^

### Scanning electron microscopy (SEM)

Condyles fixed in 2.5% glutaraldehyde at 4 °C were examined with SEM (JSM-6701F, JEOL, Tokyo, Japan) to identify ultrastructural changes at the osteochondral junction (*n* = 3). Tissue processing was performed according to standard procedures for high-vacuum SEM.

### Enzyme-linked immunosorbent assay (ELISA)

The content of norepinephrine (NE) and prostaglandin E2 (PGE2) in the serum of mice and norepinephrine (NE), prostaglandin E2 (PGE2), and neuropeptide Y (NPY) in the subchondral bone of the mice condyle were detected using ELISA kits (*n* = 3). The concentrations of subchondral bone NE (mouse ELISA kit, Elabscience, China), NPY (mouse ELISA kit, Elabscience, China), PGE2 (mouse ELISA kit, Elabscience, China) were measured individually.

### Statistical analyses

Data analysis was performed using GraphPad Prism 9.4.1. The normality of datasets and homogeneity of variances were respectively assessed using the Shapiro-Wilk test and the modified Levene’s test, respectively. Student’s *t*-test was employed to compare differences between two groups. One-way analysis of variance (ANOVA) was utilized for analyzing differences among more than two groups. Results were presented as mean ± standard deviation, with *P* < 0.05 considered statistically significant.

The experimental procedures for behavior tests, viral anterograde tracing, histochemical and immunofluorescence staining and In vitro experiments are provided in the supplementary meterials.

## Supplementary information

Supplementary Materials for Effect of regional crosstalk between sympathetic nerves and sensory nerves on temporomandibular joint osteoarthritic pain

Representative results of live cell calcium imaging in TGN group

Representative results of live cell calcium imaging in TGN+NE group

Representative results of live cell calcium imaging in TGN+PGE2 group

Representative results of live cell calcium imaging in TGN+NE+PGE2 group

## Data Availability

All data needed to evaluate the conclusions in the paper are present in the paper and/or the Supplementary Materials. Additional data related to this paper may be requested from the authors.
