# Supplementary material for: Effect of regional crosstalk between sympathetic nerves and sensory nerves on temporomandibular joint osteoarthritic pain
Source: Int J Oral Sci. 2025 Jan 7;17:3. doi: 10.1038/s41368-024-00336-6 (PMC11704193; doi:10.1038/s41368-024-00336-6)
Supplement: Supplementary file 1 — Supplementary Materials for Effect of regional crosstalk between sympathetic nerves and sensory nerves on temporomandibular joint osteoarthritic pain [file 41368_2024_336_MOESM1_ESM.docx]

Supplementary Materials for

**Effect of regional crosstalk between sympathetic nerves and sensory nerves on temporomandibular joint osteoarthritic pain**

Zhangyu Ma^1^†, Qianqian Wan^2^†, Wenpin Qin^1^, Wen Qin^2^, Jianfei Yan^1^, Yina Zhu^2^, Yuzhu Wang^2^, Yuxuan Ma^2^, Meichen Wan^2^, Xiaoxiao Han^1^, Haoyan Zhao^1^, Yuxuan Hou^1^, Franklin R. Tay^3^, Lina Niu^2*^, Kai Jiao^1*^

*Corresponding author. Email: kjiao1@163.com (K.J.); niulina831013@126.com (L.N.N.)

†These authors contributed equally to this work.

**This file includes:**

Graphic abstract. Schematic depicting the involvement of regional sympathetic nerves in the mediation of pain in early-stage TMJ-OA.

Figure S1. ELISA of serum NE and PGE2 levels in mice.

Figure S2. Intraoperative and postoperative images of superior cervical ganglionectomy.

Figure S3. Representative immunofluorescent images and quantitative analysis of TH and DAPI of the murine condyles.

Figure S4. Schematic of the immunofluorescent staining timepoint.

Figure S5. Schematic of the viral anterograde tracing in the trigeminal ganglion and the superior cervical sympathetic ganglion experiment procedures.

Figure S6. The immunofluorescence staining results of sympathetic nerves in the condyles of control group mice at different time points.

Figure S7. The immunofluorescence staining outcomes of sensory nerves in the condyles from control group mice at different time points.

Figure S8. The immunofluorescence staining results of sympathetic and sensory nerves in the condyles of control group mice at different time points.

Figure S9. The viral anterograde tracing results of sympathetic and sensory nerves in the condyles of control group mice at different time points.

Figure S10. Results of Von-Frey, open field and elevated plus maze tests at different time points after UAC induction.

Figure S11. Representative immunofluorescent staining images and quantitative analysis of DAPI (blue) and β3-tubulin (green) of the TGN group and TGN + SCGN group.

Table S1. Primer sequences used for RT-PCR.

Supplementary Materials and Methods

Supplementary Information


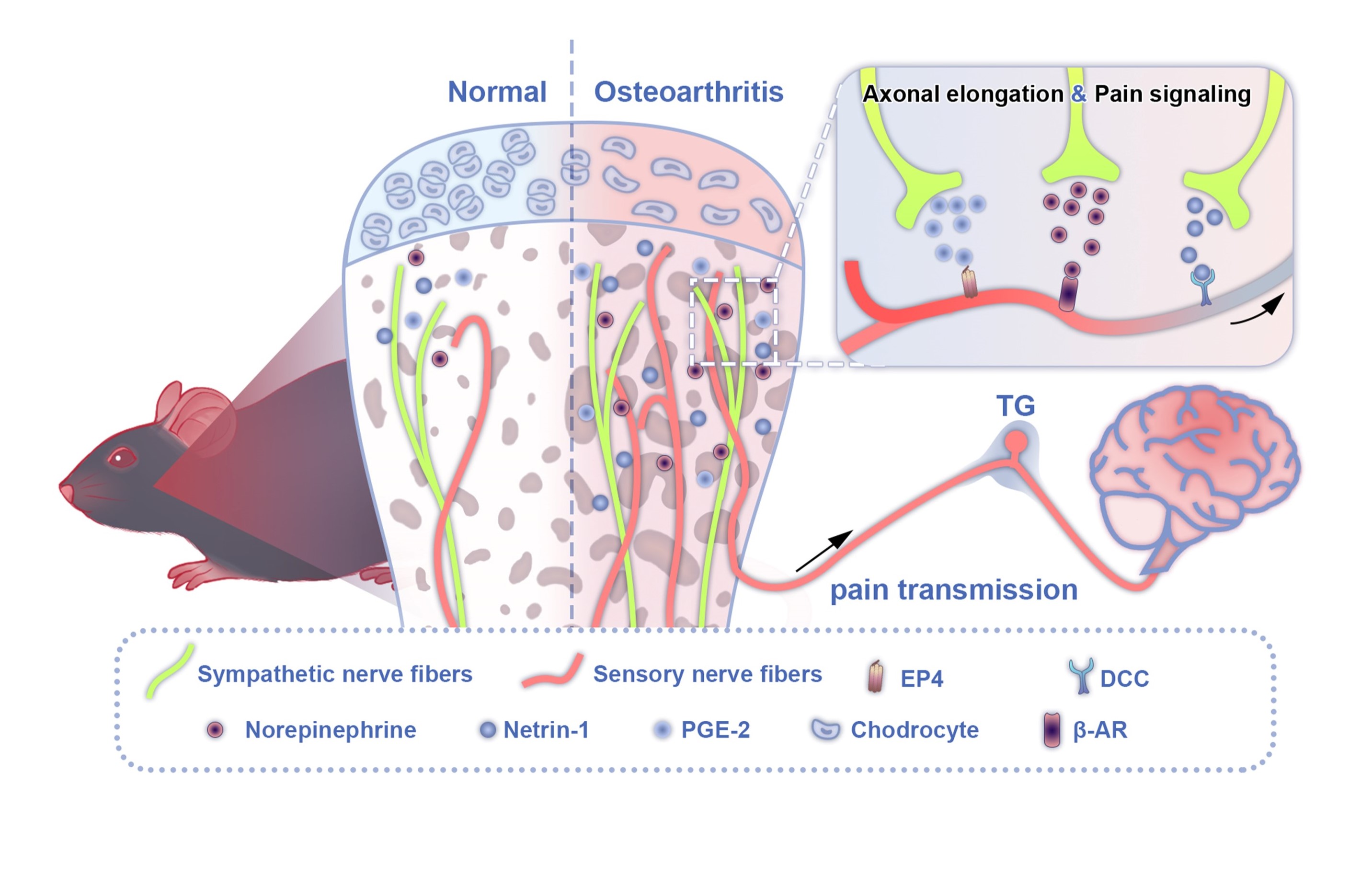


**Graphic abstract.** **Schematic depicting the involvement of regional sympathetic nerves in the mediation of pain in early-stage TMJ-OA.** Sympathetic nerves precede sensory nerves and run in parallel with sensory nerves in the subchondral bone of TMJ-OA mice. During the early stage of TMJ-OA, regional sympathetic nerves release increased levels of norepinephrine (NE). The released NE promotes growth and activation of sensory nerves in the subchondral bone. The NE also synergizes with the actions of netrin-1 and PGE2, thereby contributing to the development of pain.


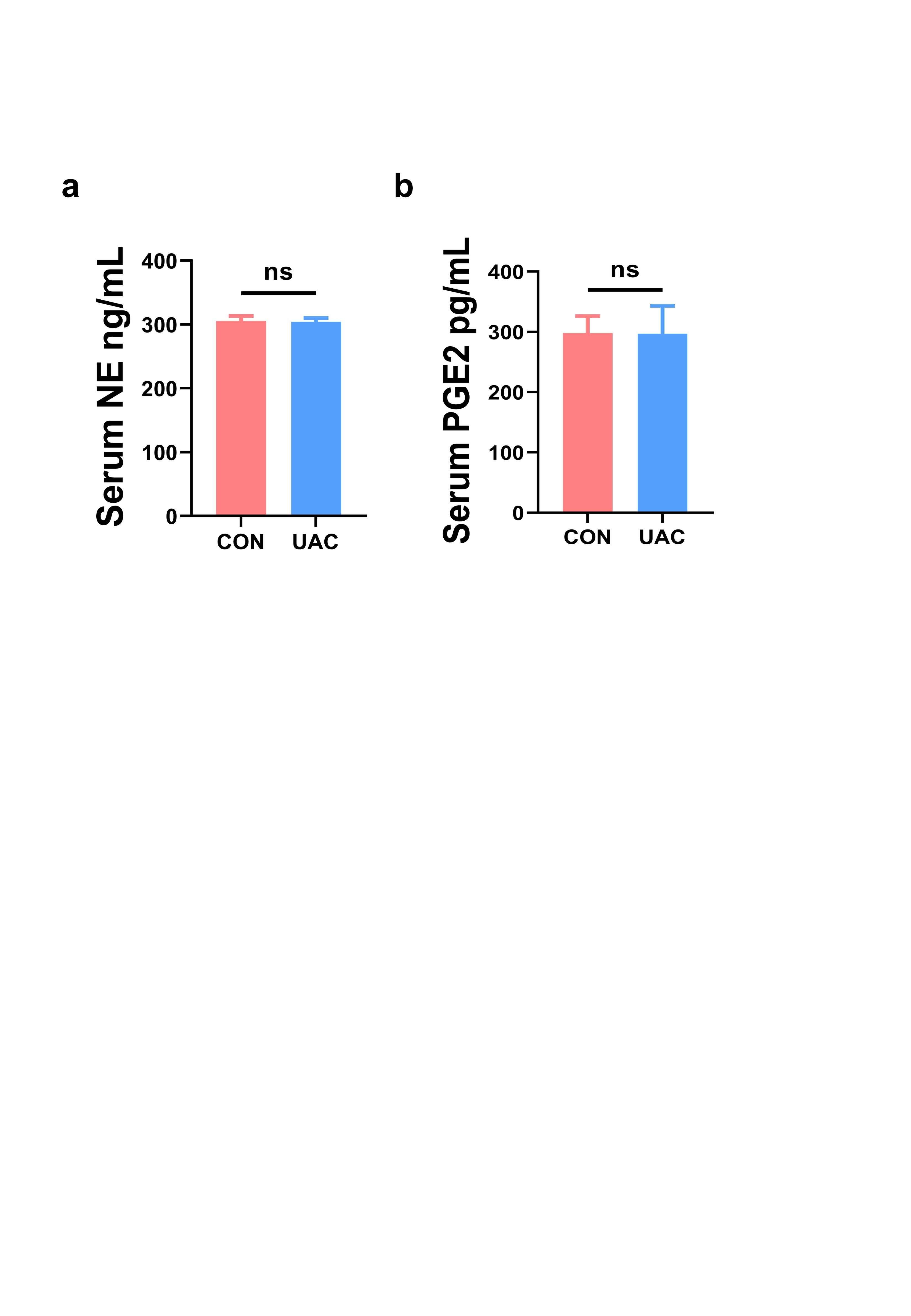


**Figure S1.** ELISA of serum NE (**a**) and PGE2 (**b**) levels in mice (*n* = 3). ns means not significant by Student’s *t* tests.


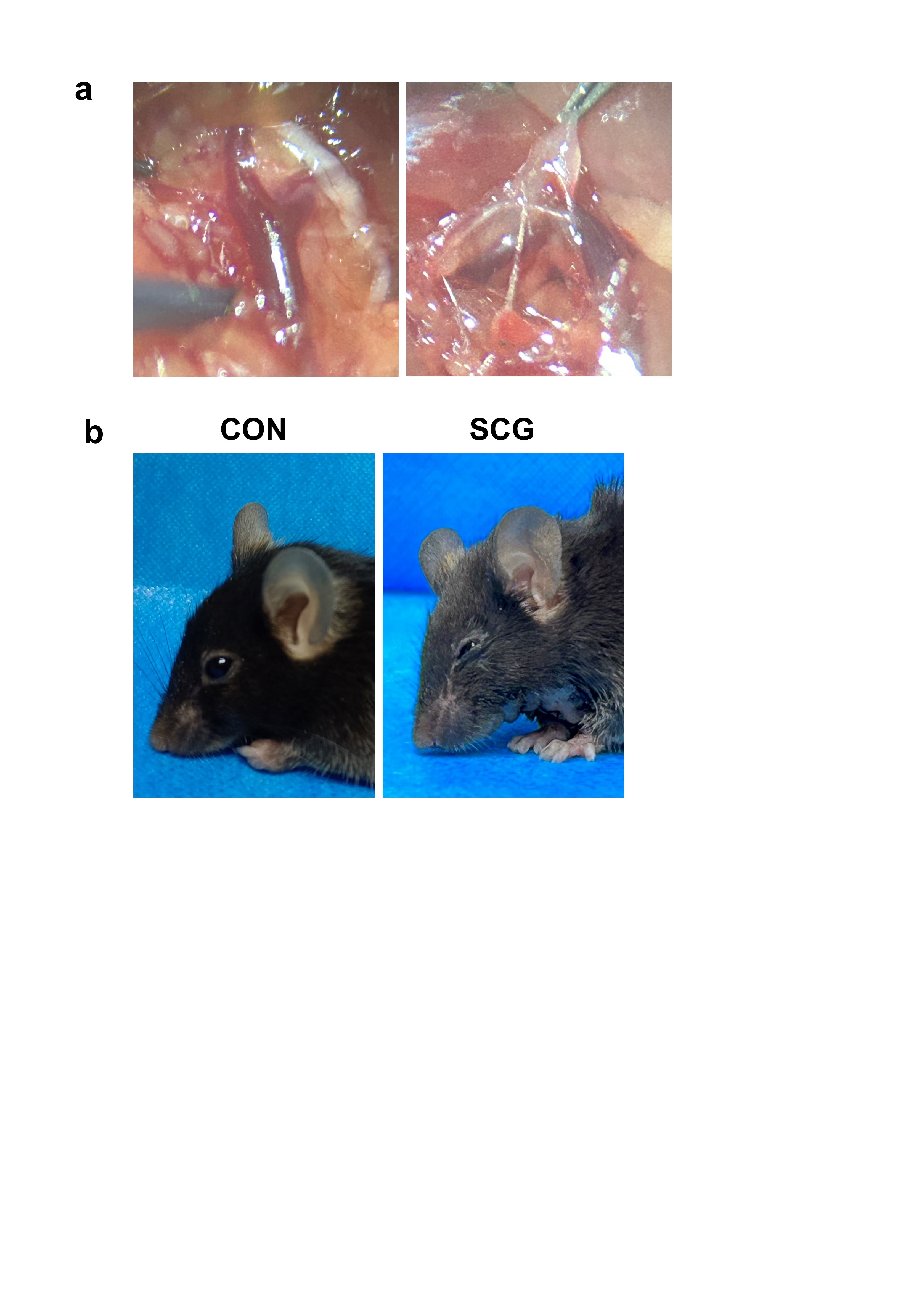


**Figure S2.** Intraoperative **a** and postoperative images **b** of superior cervical ganglionectomy.


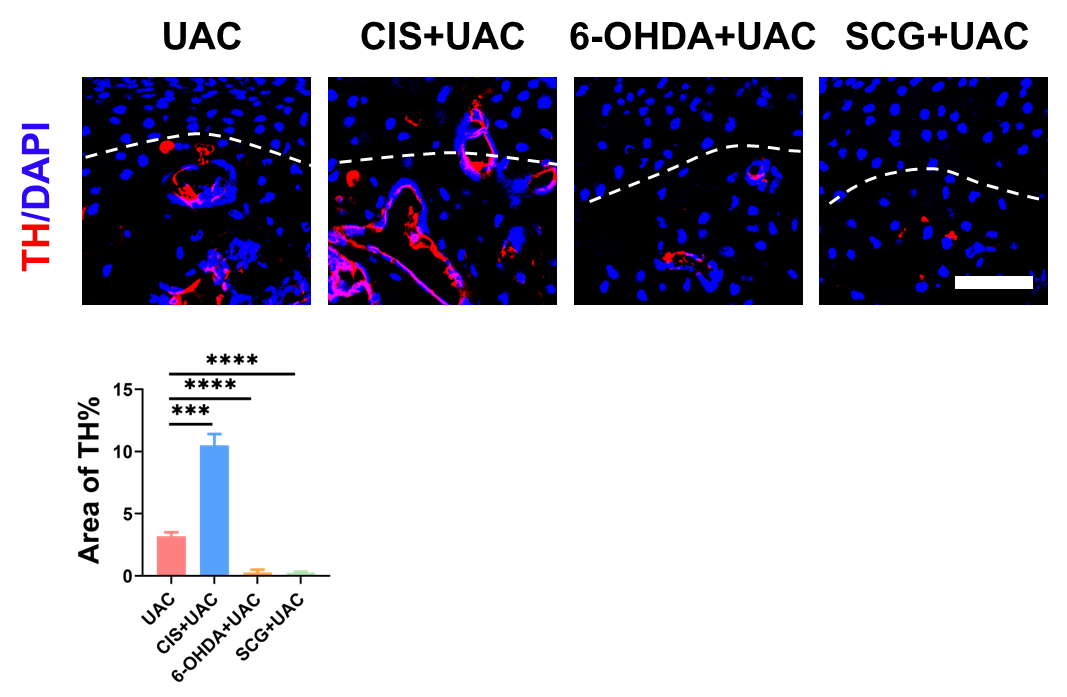


**Figure S3.** Representative immunofluorescent images and quantitative analysis of TH (red) and DAPI (blue) of the murine condyles (scale bars, 50 μm; *n* = 3). *** means *P* < 0.001, and **** means *P* < 0.0001 by one-way ANOVA.


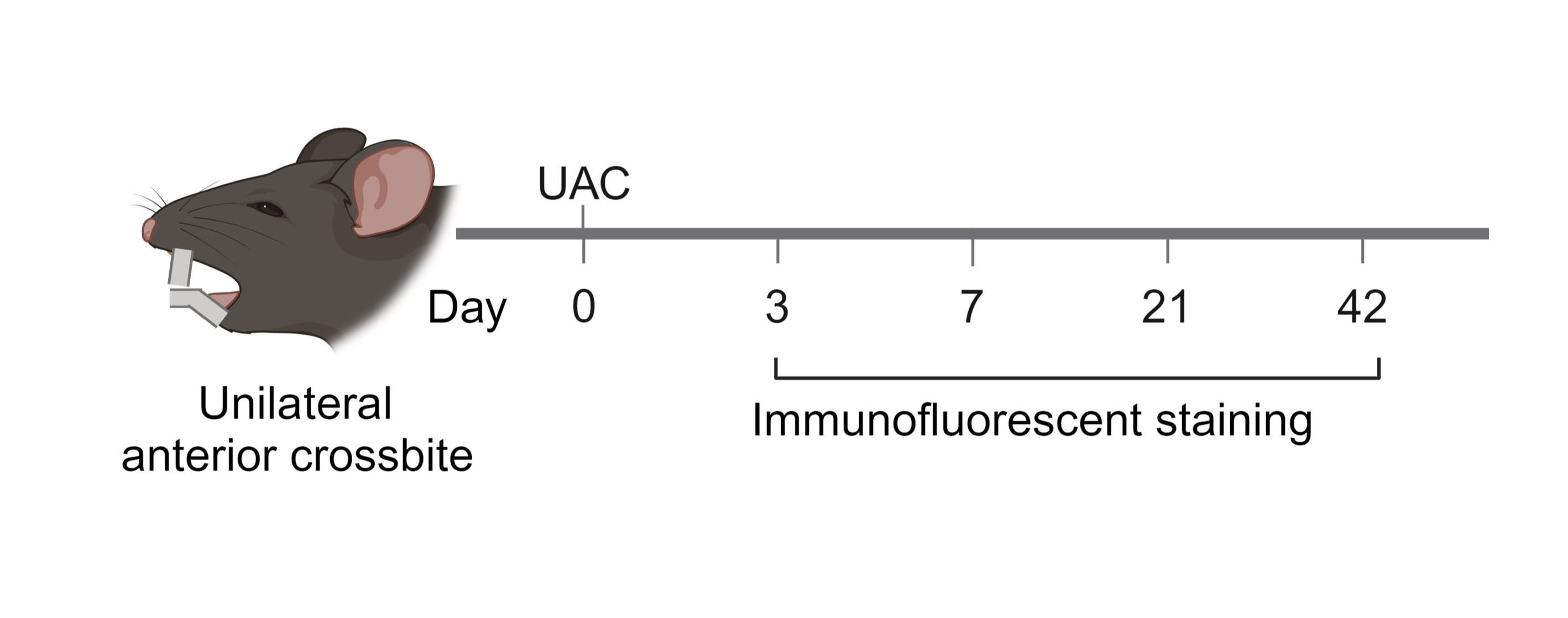


**Figure S4.** Schematic of the immunofluorescent staining timepoint. Schematic generated with Bio Render.


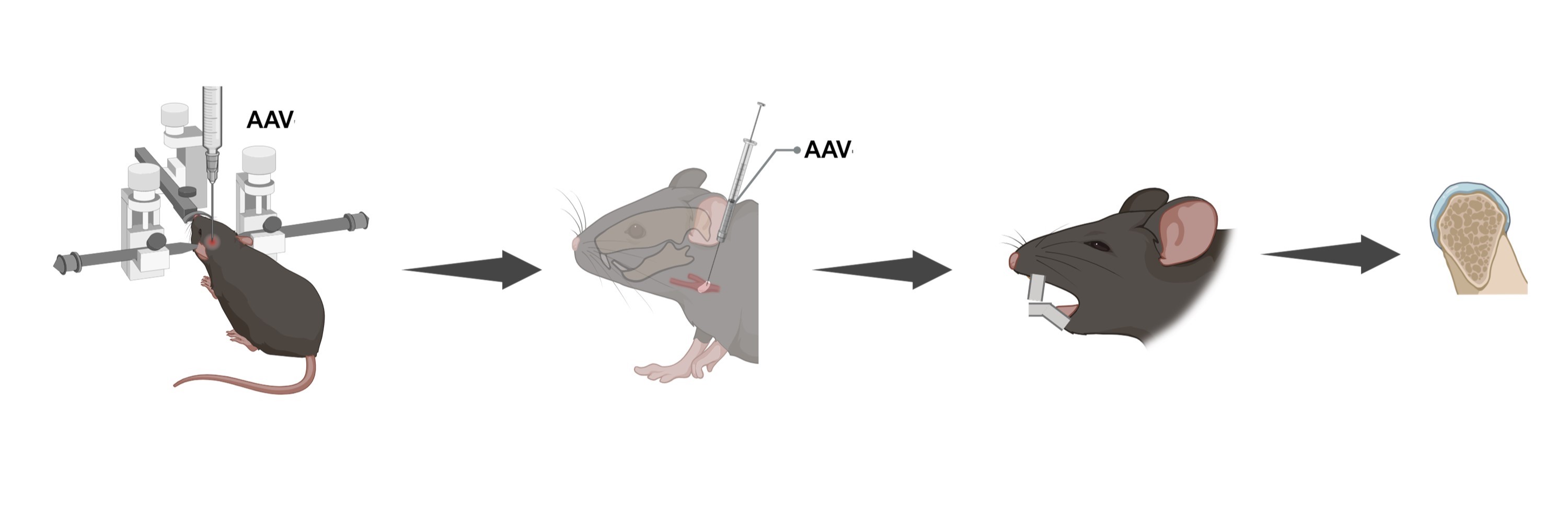


**Figure S5.** Schematic of the viral anterograde tracing in the trigeminal ganglion and the superior cervical sympathetic ganglion experiment procedures. AAV means adeno-associated virus. Schematic generated with Bio Render.


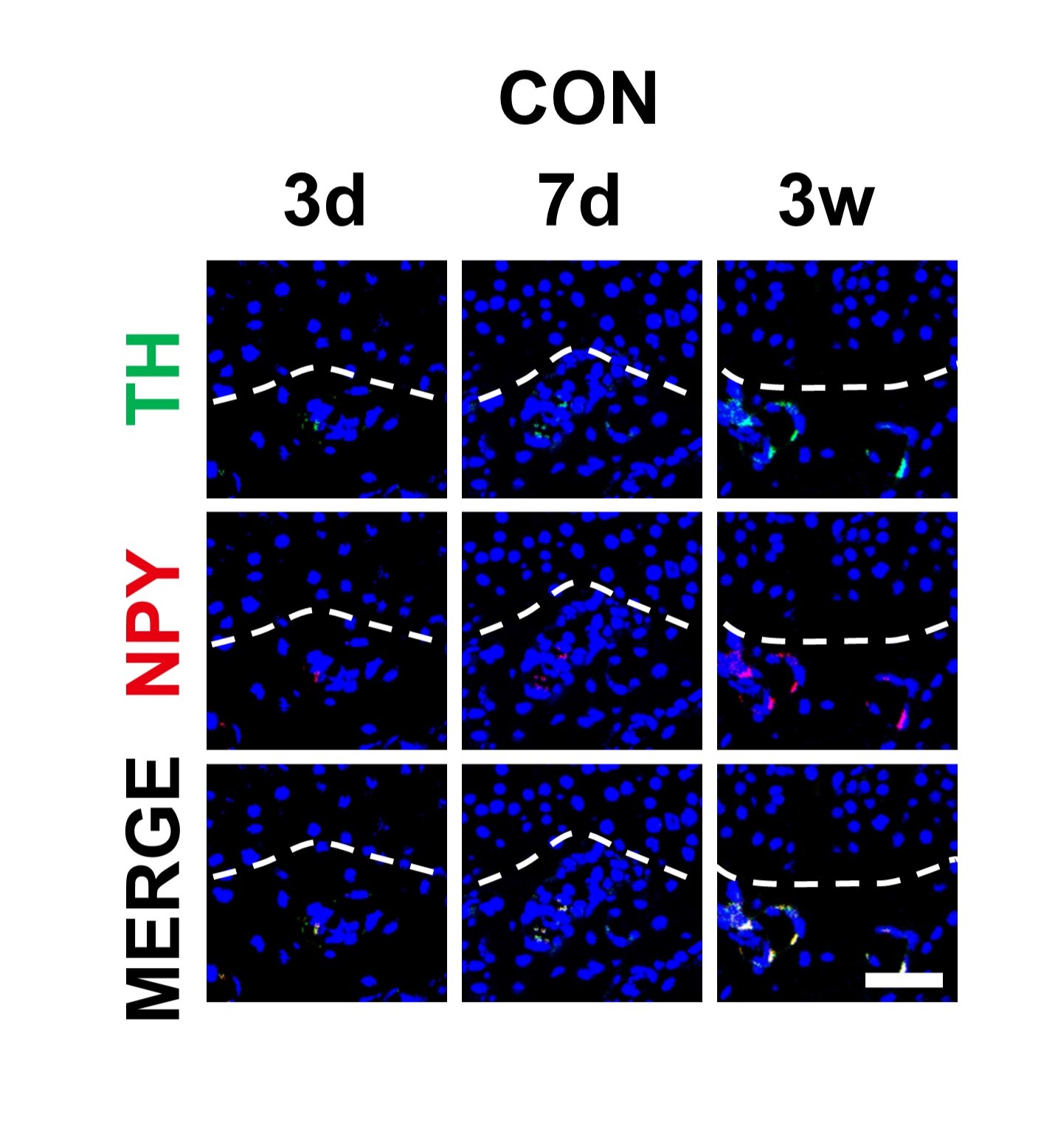


**Figure S6.** The immunofluorescence staining results of sympathetic nerves in the condyles of control group mice at different time points. Representative immunofluorescent staining images and quantitative analysis of DAPI (blue), TH (green), NPY (red) of the murine condyles (scale bars, 50 μm; *n* = 3).


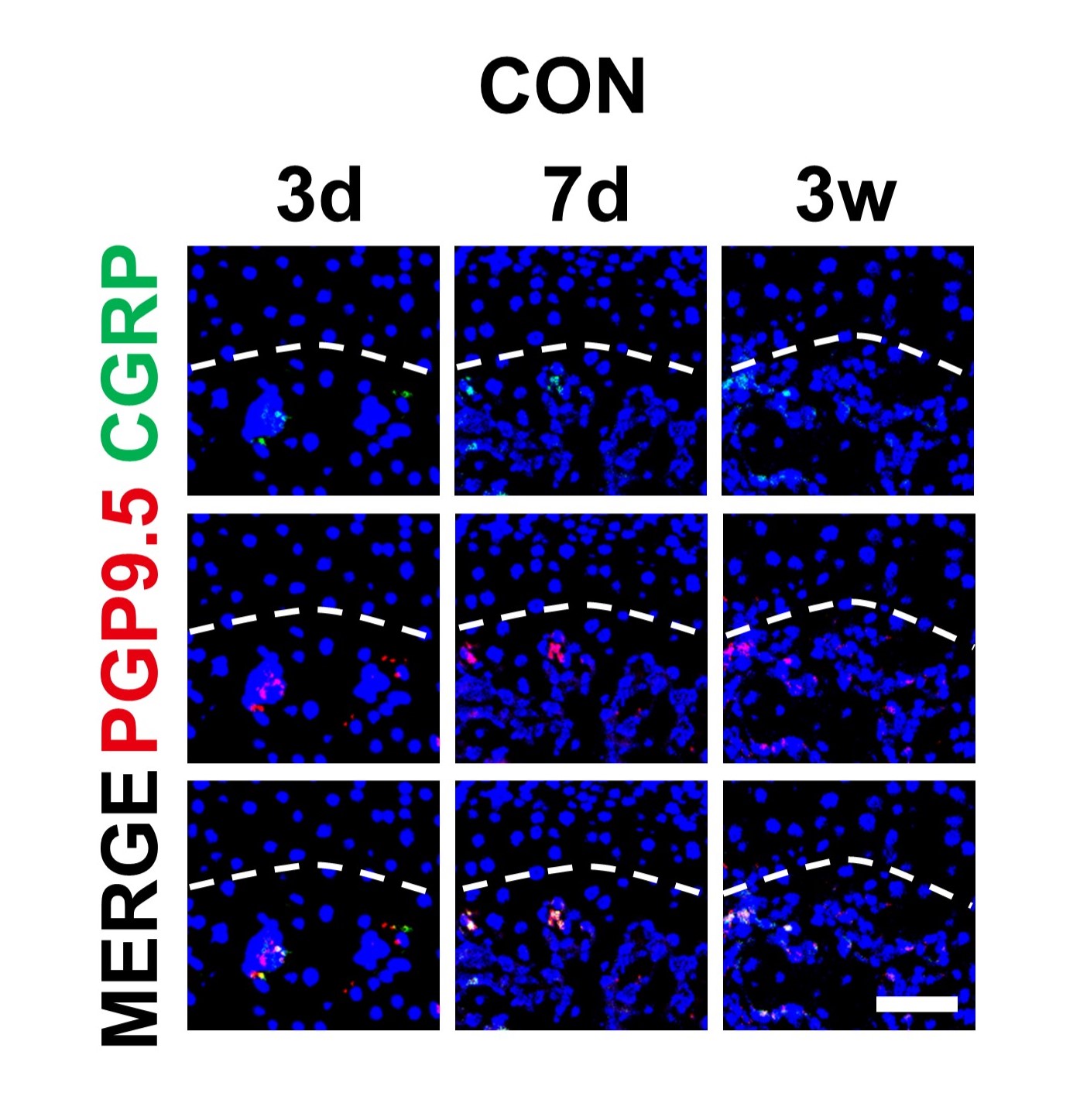


**Figure S7.** The immunofluorescence staining outcomes of sensory nerves in the condyles from control group mice at different time points. Representative immunofluorescent staining images and quantitative analysis of DAPI (blue), CGRP (green) and PGP9.5 (red) of the murine condyles (scale bars, 50 μm; *n* = 3).


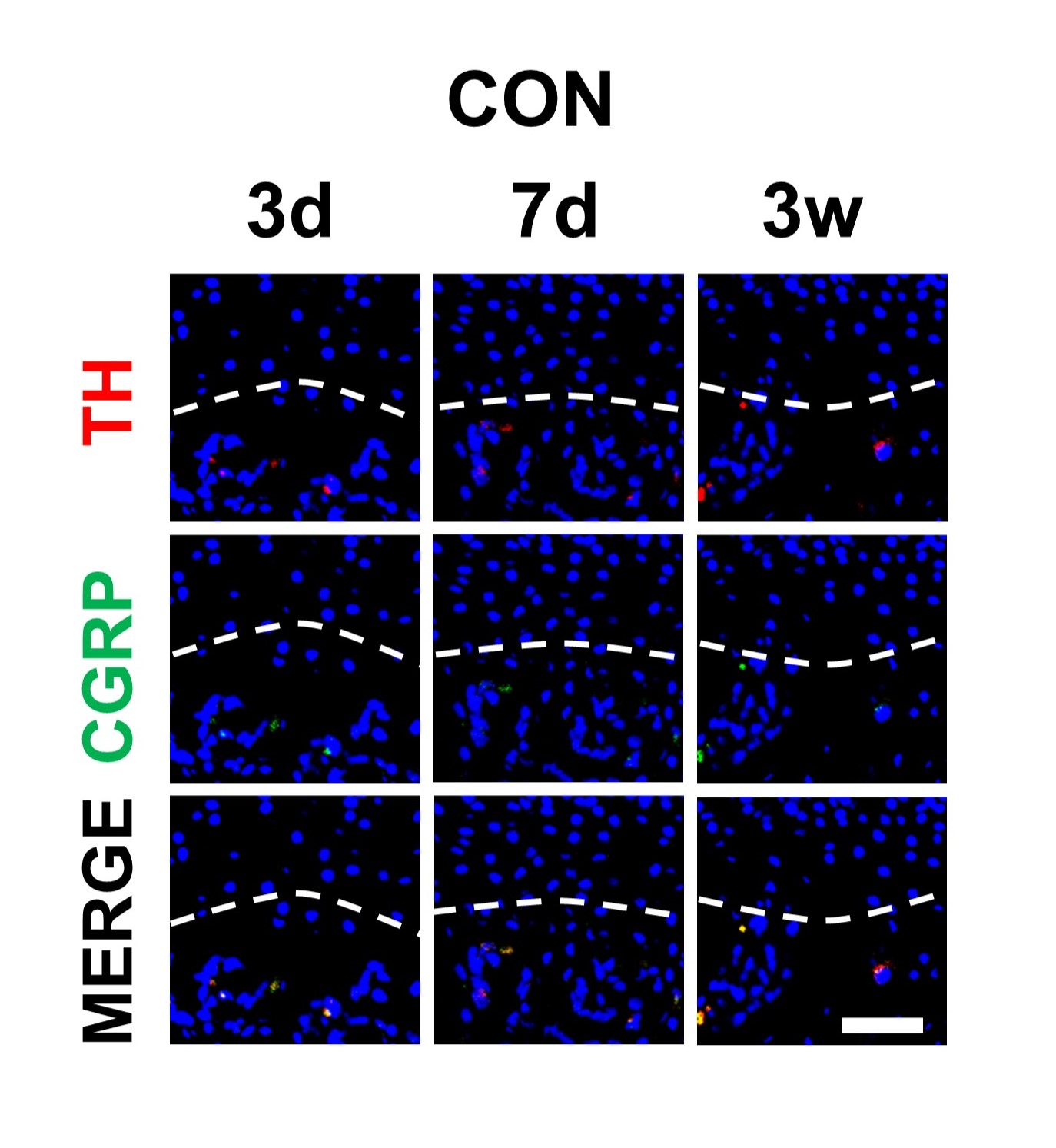


**Figure S8.** The immunofluorescence staining results of sympathetic and sensory nerves in the condyles of control group mice at different time points. Representative immunofluorescent staining images and quantitative analysis of DAPI (blue), TH (red) and CGRP (green) of the murine condyles (scale bars, 50 μm, *n* = 3).


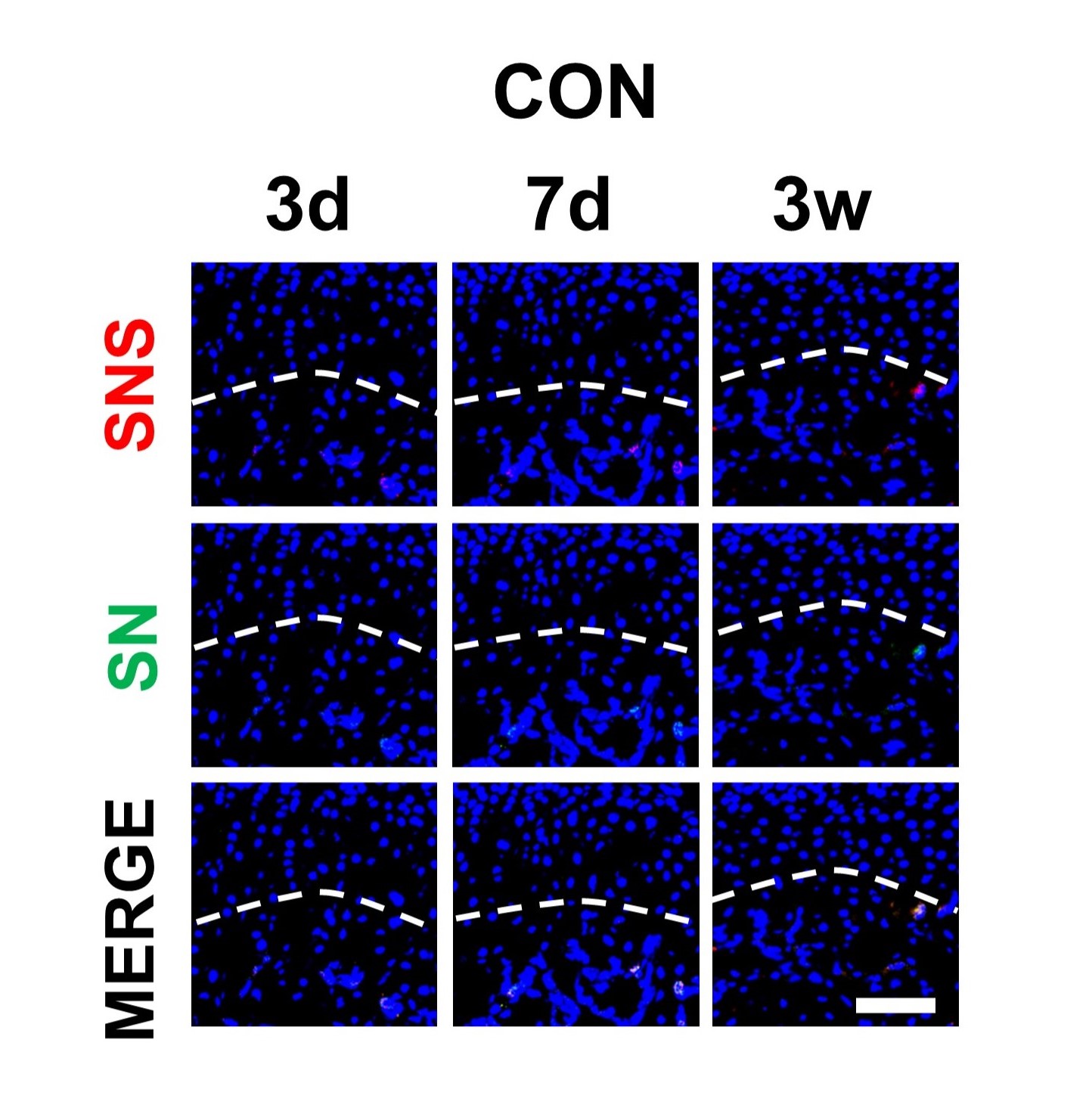


**Figure S9.** The viral anterograde tracing results of sympathetic and sensory nerves in the condyles of control group mice at different time points. Representative results, and quantitative analysis of viral anterograde tracing in the trigeminal ganglion (green) and the superior cervical sympathetic ganglion (red). Nucleus were stained with DAPI (blue). SNS means sympathetic nervous system. SN means sensory nerves (scale bars, 50 μm, *n* = 3).


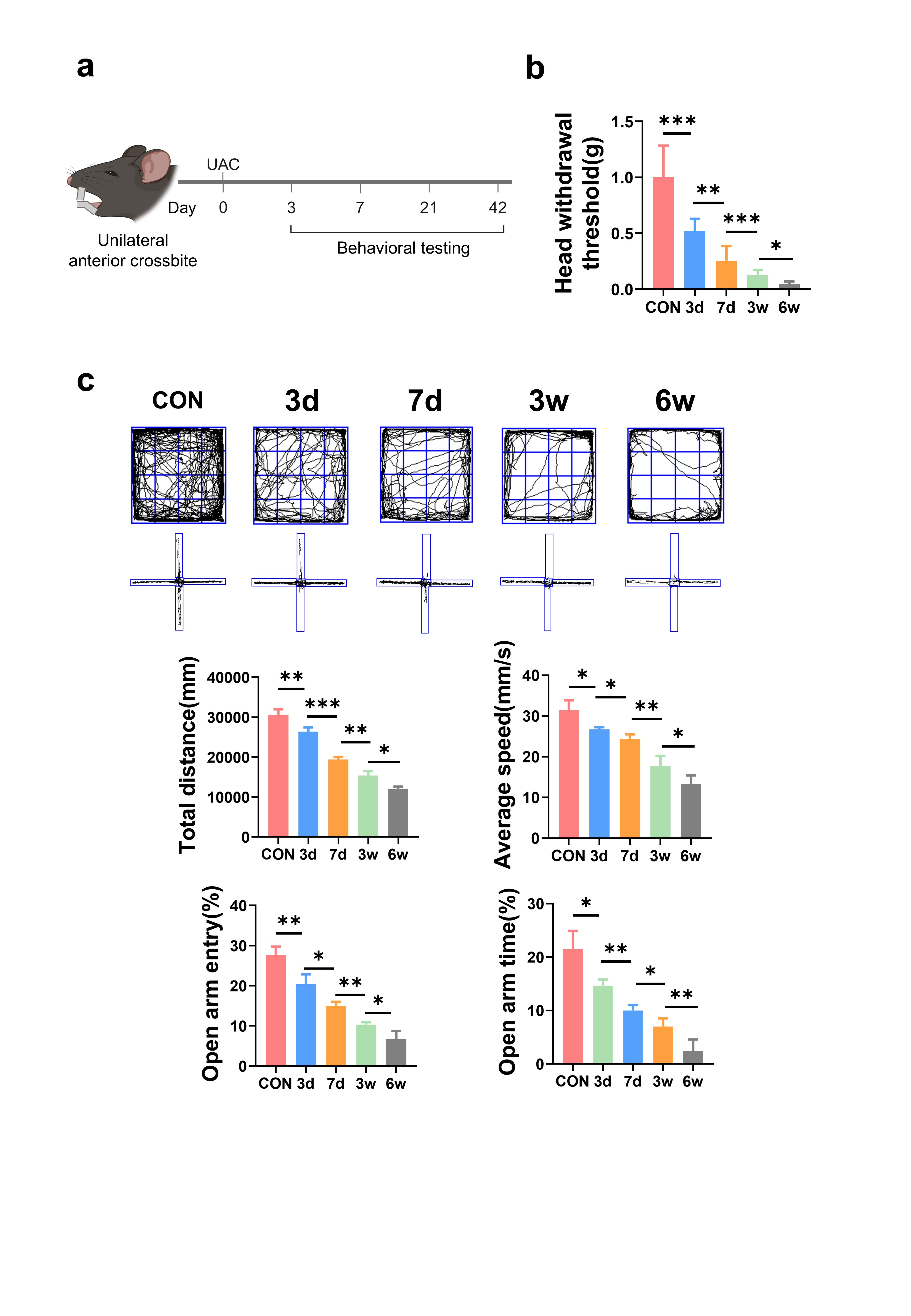


**Figure S10.** Results of Von-Frey, open field and elevated plus maze tests at different time points after UAC induction. **a** Schematic of the experiment procedures. **b** Von-frey test results of mice in different groups (*n* = 6). **c** Representative results and quantitative analysis of the open field test and the elevated plus maze test (*n* = 6). * means *P* < 0.05, ** means *P* < 0.01, and *** means *P* < 0.001 by one-way ANOVA. Schematic generated with Bio Render.


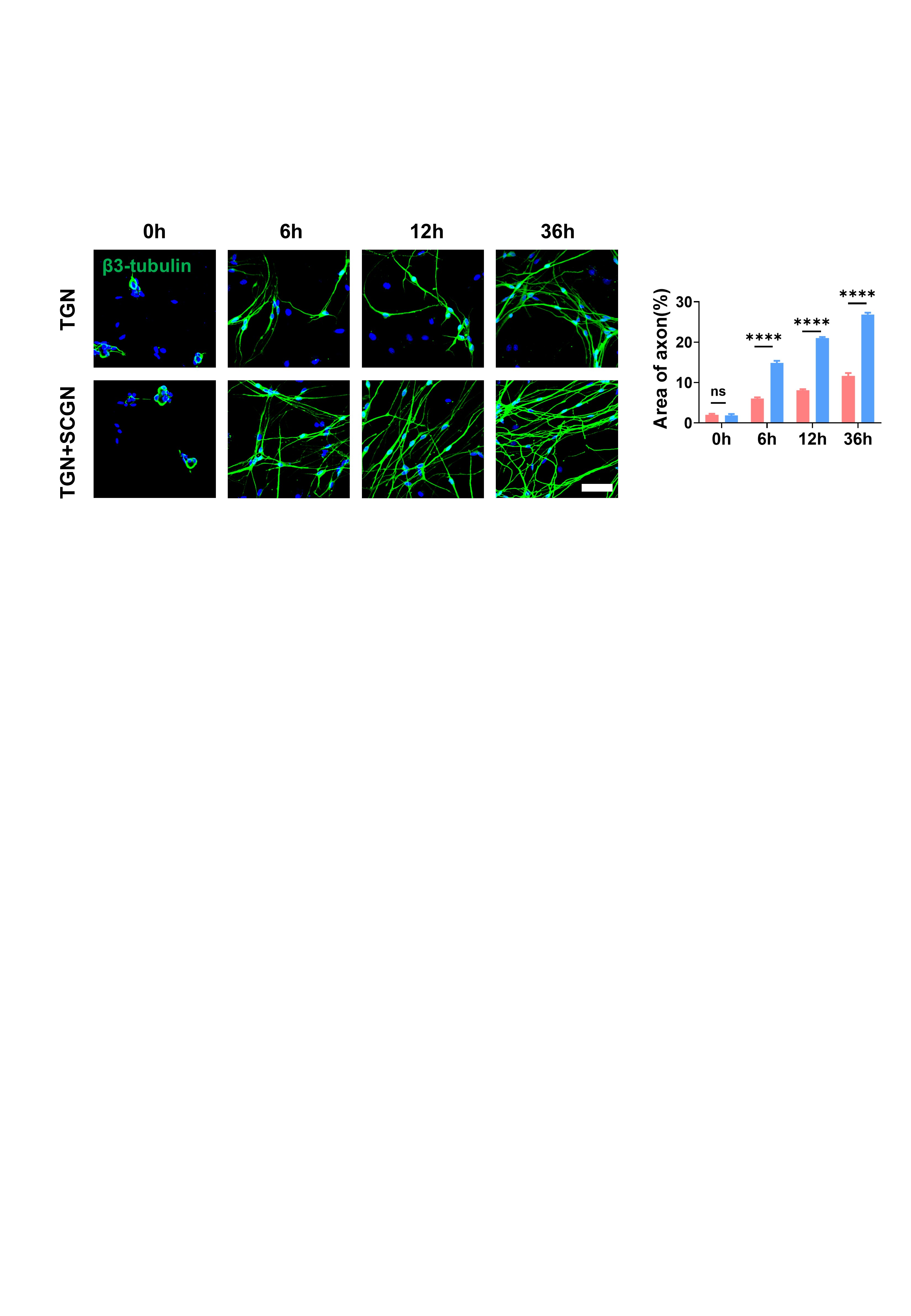


**Figure S11.** Representative immunofluorescent staining images and quantitative analysis of DAPI (blue) and β3-tubulin (green) of the TGN group and TGN + SCGN group (scale bars, 60 μm; *n* = 3). ns means not significant and **** means *P* < 0.0001 by Student’s *t* tests.

| Gene name | Sequence (5’-3’) |
| --- | --- |
| R-PACAP-F | ACGAAGCCTACCGCAAAGTC |
| R-PACAP-R | TGAAGATGCCGTCCGAGTG |
| R-CGRP-F | TCCAGGCAGTTCCTTTGAGG |
| R-CGRP-R | GCCAGTAGGCGAGCTTCTTC |
| R-SP-F | TATTGGTCCGACTGGTCCGA |
| R-SP-R | CTGCTGAGGCTTGGGTCTTC |

Table S1. Primer sequences used for RT-PCR.

**Supplementary Materials and Methods**

1. **Behavior tests**

Using a calibrated Von-frey filament (Ugo Basile, Varese, Italy), the mechanical hyperalgesia of the oral and maxillofacial region in mice from different groups was examined at various time-points (*n* = 6). Briefly, the mouse was allowed to acclimate to the experimental setting for 30 min. Then each mouse was placed into a small cage with limited space that prevented it from turning around. A force was then applied to the skin region using a Von-Frey filament. The force magnitude was adjusted such that the filament bends at an angle of 45°. Each filament exerted its force on the skin of the trigeminal nerve-innervated maxillofacial region for 1-2 s, with a 10 s interval between application. This procedure was repeated 5 times. The stimulus started with the lowest filament force (0.08 g) and gradually increased in ascending order. A positive response was recorded when the mouse rapidly withdrew its head upon stimulation. If three out of the five stimuli elicited a positive response, the force at which this occurred was considered the head withdrawal threshold.

The open field test was used to record spontaneous behavior and exploratory activity. Prior to behavioral testing, each mouse was allowed to habituate in the experimental environment for a minimum of 30 min. The mouse was subsequently placed in the central area of the open field arena, where its activities were monitored and recorded using a camera for 15 min. This process captured the movement trajectory of a mouse during its exploration within the open field.

The elevated plus maze test was used to record exploratory behavior in the mice. Prior to the experiment, each mouse was allowed to habituate in the experimental environment for at least 30 min. The mouse was then placed at the central “cross” of the maze, and their activities were recorded for 5 min. During this time, trajectories of the mice were identified. Observations were made on the number of entries into both the open and closed arms, as well as the time spent in each.

1. **Viral anterograde tracing**

**Viral anterograde tracing of the trigeminal ganglion (TG):** Recombinant self-fluorescent adeno-associated virus (AAV) was used for inoculation. The mice were anesthetized with a mixture of 2% isoflurane and oxygen, followed by deep anesthesia with pentobarbital (*n* = 6). A 2 cm long midline skin incision was made from the head to the neck, to expose the skull. Craniotomy (1 mm diameter) was performed at the corresponding coordinates for the trigeminal ganglion (X = ±1.45, Y = -1.34, Z = -5.65) using a dental drill. A glass micropipette (tip diameter 40-60 μm) was connected to a microsyringe (1 μL, Hamilton, NV, USA). One hundred nanoliter of rAAV (recombinant adeno-associated virus)-hSyn (human synapsin promoter)-EGFP (enhanced green fluorescent protein)-WPRE (enhanced green fluorescent protein)-hGH polyA (human growth hormone polyadenylation signal) was used for inoculation. This rAAV exhibits green fluorescence and was supplied by BrainVTA, Wuhan, China at a concentration of 2×10^12^ viral genomes/mL. The wound was sutured after inoculation.

**Viral anterograde tracing of the cervical superior sympathetic ganglion:** Recombinant self-fluorescent AAV was used for inoculation. The mice were anesthetized with a mixture of 2% isoflurane and oxygen, followed by deep anesthesia with pentobarbital (*n* = 6). A midline incision was made in the neck to expose the bifurcation of both common carotid arteries. The cervical superior sympathetic ganglion was then identified at its characteristic location on the posterior aspect of the artery bifurcations. Using the 1 μL capacity Hamilton syringe, 100 nL of rAAV-CAG (chicken beta-actin promoter)-mCherry-WPRE (Woodchuck hepatitis virus post-transcriptional regulatory element)-hGH polyA was injected into the cervical superior sympathetic ganglion. This rAAV exhibits red fluorescence and was supplied by BrainVTA, Wuhan, China at a concentration of 2×10^12^ viral genomes/mL. The wound was sutured after inoculation.

Mice of all groups were euthanized with an overdose of pentobarbital. Portions of the condyles were collected and fixed in 4% paraformaldehyde at 4 °C for 24 h. After fixation, the condyles were decalcified using 4% ethylenediamine tetraacetic acid at room temperature for four weeks, dehydrated in 30% sucrose for three days, embedded in optimal cutting temperature compound (Leica, Wetzlar, Germany), and stored at -80 °C. Central sagittal sections were prepared on a cryostat-supported microtome (CM1950; Leica, Germany), stained, and examined with a light microscope (Leica Microsystems, Germany). The remaining condyles were preserved at -80°C for protein extraction purposes.

1. **Histochemical and immunofluorescence staining**

Selected central sagittal sections of the condyles were stained with hematoxylin & eosin (HE), and safranin O/fast green. Stained sections (*n* = 3) were examined with a light microscope and analyzed with ImageJ software (National Institutes of Health, Bethesda, MD, USA).

For immunofluorescence staining (*n* = 3), the central sagittal sections of each condyle and cell culture slides were treated with 1% Triton X-100 (MilliporeSigma, USA), blocked with 10% goat serum (Beyotime Biotech, Inc., Jiangsu, China), and incubated overnight with primary antibodies at 4 °C. The primary antibodies used were protein gene product 9.5 (ab86808, PGP 9.5, diluted at 1:300, Abcam, Cambridge, United Kingdom), calcitonin gene-related peptide (14959, CGRP, diluted at 1:400, Cell Signaling Technology, Danvers, MA, USA), tyrosine hydroxylase (58844 and 45648, TH; diluted at 1:400, Cell Signaling Technology, USA), neuropeptide Y (ABS 028-08-02, NPY, diluted at 1:300, Thermo Fisher Scientific, Waltham, MA, USA), netrin-1 (bs-1858R, diluted at 1:300, Bioss, Inc., Woburn, MA, USA), c-fos (ab208942 and ab222699, diluted at 1:300, Abcam, United Kingdom), tartrate resistant acid phosphatase (TRAP, sc-376875, diluted at 1:300, Santa Cruz Biotechnology, Dallas, TX, USA), β3-tubulin (5568, diluted at 1:300, Cell Signaling Technology, USA), substance P (SP, sc-21715, diluted at 1:300, Santa Cruz Biotechnology, Dallas, TX, USA) and pituitary adenylate cyclase-activating polypeptide (PACAP, sc-166180, diluted at 1:300, Santa Cruz Biotechnology, Dallas, TX, USA). Samples were washed with PBS, and incubated with a secondary antibody for 1 h in the dark. The corresponding secondary antibodies (ab150077, ab150116 and ab150160, diluted at 1:400, Abcam, United Kingdom) were used. The sections were then mounted with Prolong Diamond Antifade Mountant containing 4′,6-diamidino-2-phenylindole (DAPI; Solarbio, China), and observed using confocal laser scanning microscopy (CLSM; Nikon A1R, Nikon Corporation, Minato-ku, Tokyo, Japan). Three randomly-selected, non-overlapping viewing fields were obtained for each sample. The relative fluorescence area was analyzed semi-quantitatively using ImageJ software.

1. ***In vitro* experiments**

Culture of trigeminal ganglion neurons (TGNs) involved aseptically dissecting the trigeminal ganglia from 3-day-old neonatal rats^1^. The isolated neurons were enzymatically digested using a combination of 0.1% collagenase (MilliporeSigma, USA) and 0.25% trypsin (Thermo Fisher Scientific, USA). After centrifuging at 1000 rpm for 3 min, the cells were resuspended and seeded onto cell culture plates pre-coated with poly-L-lysine at a concentration of 0.2 m/mL (MilliporeSigma, USA). Plating density was approximately 1.0 × 10^3^ cells per plate. The cultured TGNs were maintained in a neurobasal medium supplemented with 0.5 mM L-glutamine (Thermo Fisher Scientific, USA), penicillin/streptomycin (dilution at 1:100, MilliporeSigma, USA), and 2% B-27 supplement (Invitrogen, Thermo Fisher Scientific, USA) in a humidified atmosphere at 37 °C with 5% CO_2_. The culture medium was refreshed every other day.

The extraction method of superior cervical ganglion neurons (SCGN)was similar to that used for cells from the trigeminal ganglion. The superior cervical ganglion was extracted aseptically from osteoarthritis rats.

Cell culture groups were as follows: TGN, TGN + netrin-1 (1109-N1-025, 200 ng/mL, R&D Systems, Minneapolis, MN, USA), TGN + NE (10^-5^ M, GLPBIO, USA), TGN + netrin-1 + NE, TGN + SCGN (two kinds of cells cocultured via Transwell chambers with 0.4 μm; Corning Inc., Corning, NY, USA), TGN + SCGN + guanethidine (10 ng/mL, GLPBIO, USA), TGN + SCGN + propranolol hydrochloride (1 μM, MilliporeSigma, USA) (*n* = 3).

At time points of 0h, 6h, 12h and 36h after coculture, the TG neurons in TGN, TGN + netrin-1, TGN + N, TGN + netrin-1 + NE, TGN + SCG, TGN + SCGN + guanethidin, TGN + SCGN + propranolol hydrochloride groups were by immunofluorescence staining of β3-tubulin (*n* = 3). The TGN group and TGN + SCG group were examined via SEM. The cell processing was performed according to standard procedures for high-vacuum SEM (*n* = 3).

**Live-cell calcium imaging:** Fluo-4 acetoxymethyl ester (Fluo-4 AM, Yeasen, Shanghai, China), a Ca^2+^-sensitive fluorescent dye, was used to measure intracellular Ca^2+^ concentration in the trigeminal ganglion cells. Briefly, the pre-cultured trigeminal ganglion cells were retrieved from the incubator and the culture medium was discarded. Cells were washed for three times with Hank's balanced salt solution (HBSS), treated with Fluo-4, AM working solution (Yeasen, China) and incubated at 37 °C for 30 min. The cells were washed three times again with HBSS, and then covered with HBSS buffer. Changes in intracellular fluorescence were observed using a Nikon confocal microscope. Calcium imaging groups for cells include: TGN group, TGN+PGE2 group (GC15948, 100 ng/mL, GLPBIO, USA), TGN+NE group (500 μM, GLPBIO, USA), and the TGN+PGE2 + NE combined group (*n* = 3).

**Quantitative Real-Time Polymerase Chain Reaction (qRT-PCR):** qRT-PCR was performed as previously described^2^. The expression levels of substance P (SP), calcitonin gene-related peptide (CGRP) and pituitary adenylate cyclase-activating polypeptide (PACAP) were examined. Glyceraldehyde-3-phosphate dehydrogenase (Gapdh) was used as a housekeeping gene. The primer sequences are listed in Table S1. Statistical analysis was performed in GraphPad Prism 9.4.1 using the 2^–∆∆Ct^ method. Student’s t-test was used for statistical comparisons. Results were presented as mean ± standard deviation, with P < 0.05 considered statistically significant.

**References**

1. Qin, W. *et al.* Effects of Electric Field‐Modulated Conductive Hydrogel on Osseoperception and Osseointegration of Dental Implants. *Adv. Funct. Mater.* 2400256 (2024).

2. Yan, J. et al. Autophagic LC3+ calcified extracellular vesicles initiate cartilage calcification in osteoarthritis. *Sci Adv.* 8, eabn1556 (2022).
